# Program Synthesis of Sparse Algorithms for Wave Function and Energy Prediction in Grid-Based Quantum Simulations

**DOI:** 10.1021/acs.jctc.2c00035

**Published:** 2022-03-16

**Authors:** Scott Habershon

**Affiliations:** Department of Chemistry, University of Warwick, Coventry, CV4 7AL, United Kingdom

## Abstract

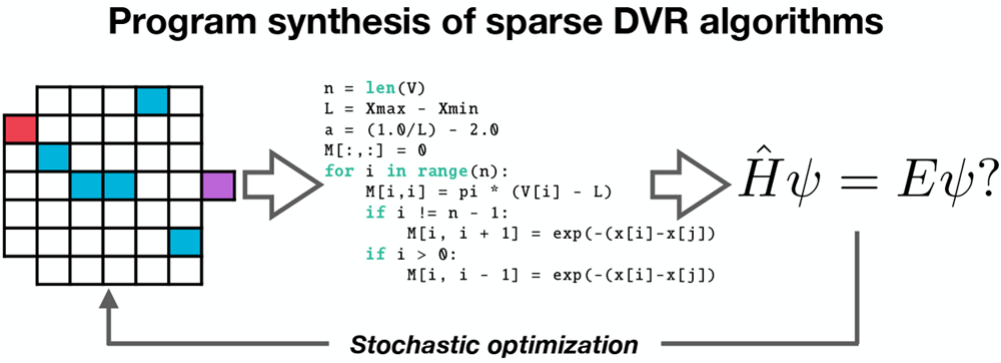

We have recently shown how program synthesis (PS), or the concept of “self-writing code”, can generate novel algorithms that solve the vibrational Schrödinger equation, providing approximations to the allowed wave functions for bound, one-dimensional (1-D) potential energy surfaces (PESs). The resulting algorithms use a grid-based representation of the underlying wave function ψ(*x*) and PES *V*(*x*), providing codes which represent approximations to standard discrete variable representation (DVR) methods. In this Article, we show how this inductive PS strategy can be improved and modified to enable prediction of both vibrational wave functions *and* energy eigenvalues of representative model PESs (both 1-D and multidimensional). We show that PS can generate algorithms that offer some improvements in energy eigenvalue accuracy over standard DVR schemes; however, we also demonstrate that PS can identify accurate numerical methods that exhibit desirable computational features, such as employing very sparse (tridiagonal) matrices. The resulting PS-generated algorithms are initially developed and tested for 1-D vibrational eigenproblems, before solution of multidimensional problems is demonstrated; we find that our new PS-generated algorithms can reduce calculation times for grid-based eigenvector computation by an order of magnitude or more. More generally, with further development and optimization, we anticipate that PS-generated algorithms based on effective Hamiltonian approximations, such as those proposed here, could be useful in direct simulations of quantum dynamics via wave function propagation and evaluation of molecular electronic structure.

## 1 Introduction

Program synthesis (PS) is a rapidly evolving technology from the field of computer science in which a central code is used to *automatically* generate new algorithms or code fragments that solve a defined problem.^1−9^ The typical inductive PS paradigm operates by optimizing an algorithm representation such that it gives the correct target outputs when presented with given inputs; in this sense, it is clear that PS bears similarities to artificial intelligence/machine-learning (AI/ML) methods,^10−29^ such as artificial neural networks (ANNs). However, the output of PS is not a set of optimized floating-point connection weights, as in typical ANN applications, but is instead a complete algorithm (often in the form of implementable code) that solves the defined input/output target problem. In addition, depending on the program structure adopted and the approach taken to code optimization, PS has the potential to propose new algorithms or solution approaches that might not necessarily have been proposed by a researcher using traditional solution methods.^3,7,8,30−32^ Furthermore, given that PS directly generates codes, rather than sets of connection weights, there is the potential that PS solutions may offer a higher level of interpretability than ANNs or similar AI/ML tools.

Recently, we have begun to explore how PS can be adapted to solve typical problems encountered in quantum chemistry.^33^ As our prototypical problem, we have previously considered the use of PS to identify algorithms that solve the one-dimensional (1-D) vibrational Schrödinger equation. In particular, we have shown that a linear code representation, in which an algorithm is represented as a set of functions operating on workspace matrices and vectors, can be used to generate algorithms (typically containing 15–25 instructions) that provide good approximations to the ground-state wave functions of vibrational Schrödinger equations for arbitrary 1-D bound-polynomial PESs. Here, a set of randomly generated PESs *V*(*x*) were represented on a uniform coordinate grid and used as PS input, with the corresponding ground-state wave functions (provided by a standard discrete variable representation [DVR] code,^34^ which similarly operates to predict eigenfunctions given as input the PES values at a set of coordinate grid points) used as target outputs. Using a stochastic instruction optimization procedure, we showed that PS can generate several new candidate algorithms to successfully solve the vibrational Schrödinger equation; importantly, we also demonstrated that these algorithms are transferrable to arbitrary bound PESs beyond those included in the input/output set used for optimization.

Upon further analysis of the resulting working equations, we found that all of the PS-generated algorithms that were generated could be viewed as variants on typical DVR algorithms.^34−41^ In particular, the ground-state wave functions were always given on output as the first eigenvector (with lowest eigenvalue) of an *n* × *n* square matrix that contained the PES *V*(*x*) evaluated at the *n* position grid-points in the diagonal elements; in addition to the PES values, further operations using the matrix indices and/or a set of problem-specific constants (such as the grid-spacing or the 1-D particle mass) were incorporated into the matrix before solution of the eigenproblem. As such, the proposed PS-generated algorithms can be viewed as approximations to an effective Hamiltonian matrix, albeit without requiring definition of an underlying set of basis functions or explicit evaluation of kinetic energy (KE) and PES matrix elements. Building on this relationship between our PS-generated codes and DVR schemes, we subsequently demonstrated that one can generate algorithms that give good approximations to the first few eigenstates of bound PESs, and we also showed that our new DVR-like schemes can be extended to multidimensional problems (with more than one active degree of freedom) in the same way that standard DVR schemes can.

The purpose of this Article is to expand on our initial proof-of-concept PS study^33^ to investigate algorithms that can predict both the allowed eigenstates *and* eigenvalues (i.e., energies) in novel DVR-type schemes applicable to multidimensional vibrational Schrödinger equations. As discussed below in section 3, determination of the energy eigenvalues in PS schemes is not as straightforward as the prediction of eigenstates alone; we investigate why this is the case and propose new PS-based approaches that can address this problem. Perhaps most importantly, we demonstrate that PS can be used to generate accurate DVR algorithms with numerically favorable matrix structures (i.e., tridiagonal structure and improved sparsity,^42^ such that an increased number of matrix elements are zero), providing an interesting alternative route toward efficient large-scale computations of wave functions for multidimensional problems, as shown below.

## 2 Theory

The general PS system employed here is the same as reported in our initial investigation;^33^ as such, we only provide a brief overview here, before focusing on the improvements relevant to the new results in section 3.

### 2.1 Problem Definition and Representation

Our target problem of interest is solution of the time-independent Schrödinger equation (TISE) to give the allowed wave functions ψ_*j*_ and corresponding energy eigenvalues *E*_*j*_.^35^ The TISE is
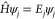
1where *Ĥ* is the Hamiltonian operator. Here, we assume that the coordinates **q** defining the system of interest form a system of orthogonal coordinates, such that the Hamiltonian operator is a sum of kinetic energy (KE) and PES contributions given by
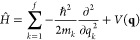
2where *m*_*k*_ is the mass associated with the degree of freedom (DOF) *k* and *V*(**q**) is the adiabatic PES describing the system of interest.

In this Article, we focus exclusively on adiabatic PESs, which are bound in all DOFs; for example, this problem setup is representative of the challenge of evaluating the eigenstates and eigenvalues associated with vibrational motion on a molecular ground-state PES. In this problem setting, an extremely common approach to solving eq 1 is the DVR scheme. In the 1-D case, diagonalization of the position operator matrix expressed in an underlying set of basis functions (typically orthogonal polynomials or sinusoidal functions) yields a grid of discrete positions, {*q*_*i*_}_*i*=1_^*n*_*g*_^, where *n*_*g*_ is the number of grid points. These grid points can be viewed as highly localized basis functions that can be used to represent the solutions of eq 1.^34−41^ The important consequence of this transformation is that PES matrix elements in this grid-localized basis are trivial to evaluate, corresponding to simply the value of the PES at the grid point, such that the Hamiltonian operator can be written in the grid-localized DVR basis as
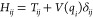
3where *V*(*q*_*i*_) is the value of the PES evaluated on grid point *q*_*i*_ and *T*_*ij*_ is the KE matrix element (obtained by analytical evaluation in the underlying polynomial basis and transformation to the grid representation). Diagonalization of the Hamiltonian matrix in eq 3 then yields the corresponding eigenfunctions and eigenvalues, with the number of grid points *n*_*g*_ controlling convergence toward the numerically exact result.

The simplicity of the DVR method, as well as the requirement that one only needs to know the value of the PES evaluated at the *n*_*g*_ grid points, makes it attractive as a method for interrogating vibrational properties and quantum dynamics of molecular systems. Typically, working in normal-mode coordinates (or similar orthogonal coordinate system) such that the KE operator is separable, one can construct a Hamiltonian matrix representation using the direct product of all grid points along each DOF; as such, the DVR scheme of eq 3 can be directly applied to multidimensional systems, enabling analysis and prediction of quantum-mechanical properties for multiple coupled vibrational modes in molecules. For example, in a 2-D system [using the explicit notation (*q*_1_,*q*_2_) = (*x*,*y*)], the DVR Hamiltonian matrix elements can be written as
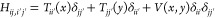
4where (*i*,*j*) and (*i*′, *j*′) label the indices of the grid point along each DOF (*x*,*y*), and there are now a total of *n*_*g*_^2^ grid points (where *n*_*g*_ is the number of grid points available along each DOF; we assume, for simplicity, that this number is the same along each DOF, but note that this is not a strict requirement). Moving to 3-D [with (*q*_1_,*q*_2_,*q*_3_) = (*x*,*y*,*z*)], the DVR Hamiltonian matrix elements are
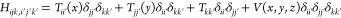
5where, again, (*i*, *j*, *k*) and (*i*′, *j*′, *k*′) label the grid points along coordinates (*x*, *y*, *z*). Of course, this approach can be extended to higher-dimensional systems following directly from eqs 4 and 5.

In DVR calculations such as those described above, it should be clear that the number of grid points scales as *n*_*g*_^*f*^, where *f* is the number of DOFs in the target problem (and assuming the same number of grid points in each DOF); given that a typical value for *n*_*g*_ is in the range of 20–50, it is clear that the problem size that can be treated with DVR schemes is limited by the requirement to evaluate the PES at all *n*_*g*_^*f*^ grid points (which could be prohibitively expensive if one requires accurate ab initio energy evaluations), as well as the requirement to manipulate increasingly large matrices. Later, we discuss how PS-generated codes can directly contribute to reducing the computational effort associated with manipulating the large matrices often encountered in DVR calculations.

At this point, it is worth making the connection between our previous PS work and the DVR algorithms discussed above. In our original PS approach,^33^ we mimic the underlying structure of DVR methods by assuming that the PES and wave function are represented on a coordinate grid of *n*_*g*_ points; as described below, the PES is provided as an input to our PS scheme, and the aim is to predict the expected output wave functions by identifying algorithms that operate on workspace matrices and vectors. Notably, we found that a universal operation in our PS-generated algorithms was the prediction of wave functions for a given PES by diagonalization of a workspace matrix **M**, such that **M** can be viewed as an approximation of a Hamiltonian-type matrix **H**, albeit without reference to any underlying basis functions. This comparison between the DVR Hamiltonian matrix and the workspace matrices generated in our PS approach will be an important aspect in discussions below.

Given the analogy between our PS strategy and well-known DVR schemes, a key hypothesis of this Article is that PS could be used to tackle the computational challenges of DVR calculations, in particular by seeking DVR-type algorithms with preferred (i.e., computationally beneficial) matrix structure. Our results in this direction are described in section 3; first, we describe our PS implementation and highlight modifications which aim to provide more efficient DVR-type schemes. Importantly, we emphasize that our PS-generated codes can be used in multidimensional problems in just the same way that DVR can be expanded to multidimensional PESs; this will be confirmed and explored later, where we discuss the computational benefits of PS-generated codes in more challenging DVR calculations.

### 2.2 Code Representation

The overall strategy adopted in our PS approach is to treat the challenge of identifying new algorithms to solve eqs 1 and 2 as an exercise in discrete optimization.^7−9^ Here, we are seeking to find an algorithm that uses, as input, the set of PES values evaluated on the coordinate grid, and gives an output vector **w**, which represents the algorithm’s prediction of the (ground- or excited-state) wave function on the coordinate grid.

Using a linear code representation, similar to that employed in Cartesian genetic programming,^7^ in which a sequence of *N* functions operate on an input workspace matrix **M** and corresponding vector **y**, a given algorithm can be encoded as a list of *N* integers labeling the operations to be performed at each algorithm step, resulting in an output vector **w** for each input problem. For a given algorithm (i.e., integer sequence), the performance can be evaluated by assessing the accuracy of the predictions given for a series of target examples for which the exact answer can be calculated using an “oracle” code. As shown in our previous work, and below, we note that the oracle code does not need to provide an excessively large number of correct input/output examples; in our experience to date, PS generally seems to operate efficiently in finding accurate, general-purpose algorithms with ∼5–20 input/output pairs.

**Figure 1:**
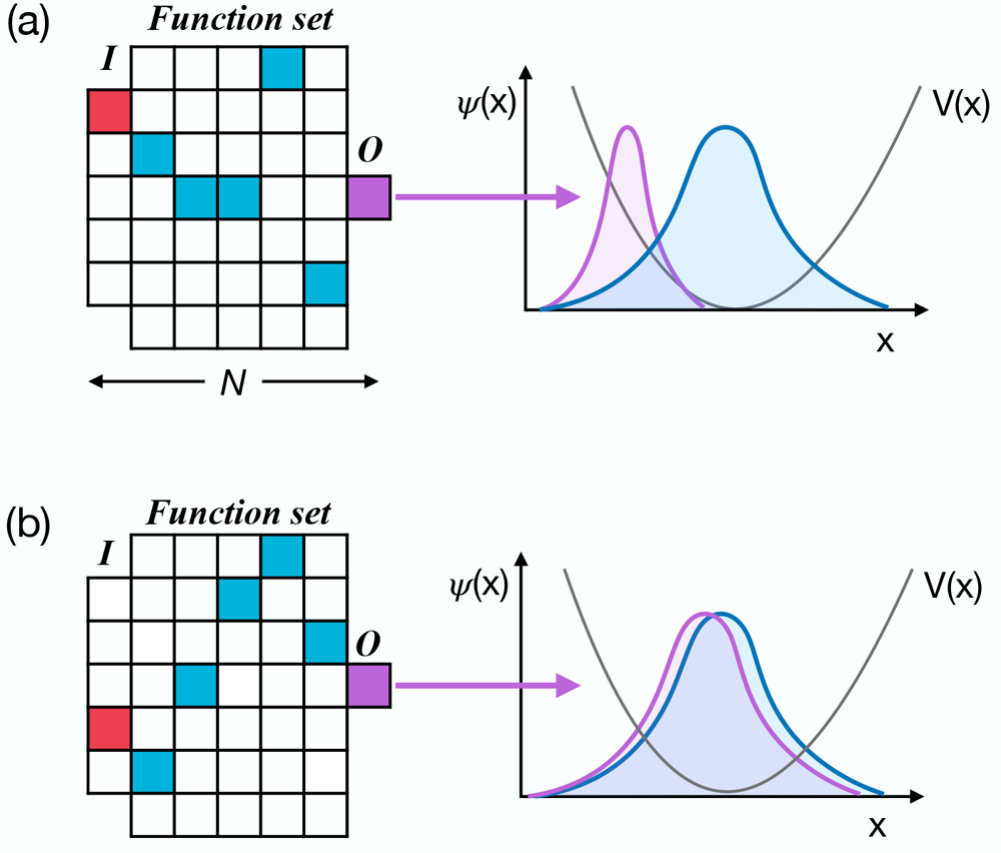
Schematic representation of PS for generating DVR-type algorithms.^33^ The grids in panels (a) and (b) show diagrammatic representations of the linear code setup employed here; at the input layer **I** and each of the internal code layers (of which there are five in the example here, represented by the five columns of the grids, excluding input and output), a choice of function is made. In the input layer, the functions define possible choices for input workspace matrices **M** and vectors **y**; in addition, the input layer defines problem specific information available to the rest of the functions, such as definitions of the PES, particle mass, and coordinate grid. In the internal layers, these objects **M** and **y** are subsequently modified by a set of function options; in the schematic example given here, there are seven function choices at each internal layer. Finally, an output function **O** gives the prediction of the corresponding wave function(s) and/or energy eigenvalues for the input PES; this can be compared to the correct target wave functions, and the code can subsequently be improved by changing the sequence of operations. Panels (a) and (b) give two representative examples of different algorithms, with panel (a) yielding poor performance and panel (b) providing good approximation of the correct target wave functions.

This approach is highlighted in Figure 1, which illustrates two schematic representations of different proposed algorithms. Each algorithm is represented by a sequence of *N* instructions, including an input instruction (**I**), an output instruction (**O**), and *N* – 2 internal code layers. At each code layer (input, output and internal), there are a number of different possible mathematical operations that are defined in a function library, as discussed below. As shown in Figure 1, a sequence of *N* instructions corresponds to a unique algorithm, starting with definition of an input workspace matrix and vector, moving on through *N* – 2 internal instructions and, finally, using an output operation to create the algorithm output; the entire sequence can be simply encoded as a set of *N* integers, defining the operation index at each of the *N* algorithm layers. We note that the same set of *n*_*i*_ instructions are used at each internal code layer in our current approach.

An important part of this PS strategy is the definition of the input, internal, and output function sets. In this Article, following our previous study, we use a large set of input and internal functions to provide flexibility in the definition of the input workspace vector **y** and matrix **M**, and a wide range of mathematical variation in the internal function set. However, given the analogy between DVR algorithms and the approximation of a Hamiltonian-type matrix via the workspace matrix **M**, in this Article, we only use a single output operation (Figure 1) corresponding to diagonalization of the workspace matrix **M** and interpretation of the corresponding eigenvectors as the predicted wave functions for the input PES. This strategy of treating the workspace matrix as an approximation of a Hamiltonian-type matrix is justified on the basis of our previous study, which found that successful algorithms generated by PS for approximating ground- and excited-state wave functions always used the calculation of eigenvectors of the workspace matrix **M** as the output operation (despite several alternative output functions being available).^33^

The set of input possibilities and internal functions used in this Article are given in the Supporting Information. Generally, these functions are similar to the set of functions used in our previous work, with the addition of a few new operations added in order to provide some additional flexibility in the algorithms which can be generated by PS.

The input functions define the workspace vector **y** and workspace matrix **M**. In keeping with the analogy with DVR algorithms, and as noted above, for a PES defined at *n*_*g*_ grid points, the vector **y** has length *n*_*g*_ and the matrix **M** is square, with size *n*_*g*_ × *n*_*g*_. We define a set of 11 input options, which are designed to “boot-strap” **y** and **M** with different possible functional forms. For example, as shown in the Supporting Information, one input option simply defines *y*_*i*_ = 1 for all elements *i*, and *M*_*ij*_ = 1 for all matrix elements (*i*, *j*). Beyond defining numerically sensible input matrices, the challenge of identifying the “best” set of input options is itself an optimization problem (as discussed in section 4).

The set of internal functions that operate on **y** and **M** through the *N* – 2 internal code layers are also somewhat arbitrary in our current PS approach. As shown in the Supporting Information, our approach is to simply define a large number of possible internal functions, and to use the same set of function options at each internal code layer. Generally, we employ function options that are often encountered in the mathematics of differential equations or in well-known methods for solving eq 1. We note here that the automated evolution of better functions is something that could be considered in the future, for example, using a similar approach to the automatically defined functions (ADFs) proposed in the context of genetic programming.^43,44^

In total, the PS simulations reported below use 134 function options at each internal code layer. These functions generally are “local”, in the sense that the operations performed on element *i* in **y** is dependent only on the index *i* or the position of the corresponding grid point, and the operations performed on element (*i*, *j*) in **M** are similarly dependent only on the indices or grid points of elements *i* and *j*. The focus on local operations in the internal function set is driven, in large part, by pragmatism; defining and coding function options that are dependent only on local matrix elements is simpler than defining nonlocal functions. Of course, we note that nonlocal functions could equally be incorporated into the PS described here, but we leave that expansion to future work; furthermore, we note that even using the limited set of functions employed here, we can generate accurate and efficient algorithms for predicting wave functions.

As well as defining the set of functions available within each code layer, our PS strategy also uses a set of constants that are available and accessible to relevant functions at all code layers. In particular, we define the set of constants **c** = [*m*, 2, 3, 4, π, *L*], where *m* is the mass associated with the 1-D degree of freedom and *L* is the range of the coordinate grid. In the calculations below, which consider systems with unit mass in each degree of freedom and fixed total grid-length *L* = 10, then we have **c** = [1, 2, 3, 4, π, 10]. These constants can be used by many of the functions defined in the internal function set, as shown in Supporting Information; for example, one allowed function is the addition of a given constant to all elements of the matrix,
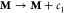
and similar operations are defined for **y**. It is worth emphasizing that there are no optimizable floating-point constants in our PS strategy, a key point in enabling generation of new algorithms that are applicable across a range of different PES functions; as such, there is a clear distinction between the typical operation of ANNs and the PS strategy used here. Finally, we note that, in addition to the constants, two further vectors, **V** and **x**, are available to all relevant input and internal functions; the vector **V** contains the PES values evaluated at all grid points for the target problem, whereas **x** contains the grid-point coordinates themselves. As shown below, and in the Supporting Information, these vectors can be used by input and internal functions to build up functional complexity in seeking more accurate matrix approximations, and can be viewed as problem definitions providing information about each individual input PES to the PS system.

### 2.3 PS by Stochastic Optimization

With the description of an algorithm as an integer sequence defining instructions at a set of *N* code layers, as well as a definition of the input, internal, and output function sets, the final aspect of PS to discuss is the optimization process; in other words, how do we identify function sequences corresponding to accurate algorithms to reproduce wave functions that obey eq 1?

To achieve this goal, we use simulated annealing (SA).^42^ In the initial implementation of our PS strategy, we defined a cost function as
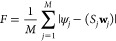
6where ψ_*j*_ is the *ground-state* wave function (represented on the coordinate grid) for the *j*th example PES, **w**_*j*_ is the current algorithm’s output vector, and *S* = ±1 is an overall sign-value chosen independently for each of the *M* target examples to give the best agreement between ψ_*j*_ and (*S*_*j*_**w**_*j*_). The factor *S*_*j*_ accounts for the fact that both +ψ and −ψ are both typically valid solutions of eq 1. Note that our initial proof-of-concept study also demonstrated that the cost function of eq 6 can be modified to also enable accurate determination of excited-state wave functions too, by simply redefining the optimization to include these additional states:
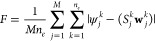
7Here, *n*_*e*_ is the number of target states included for each input target PES, with ψ_*j*_^*k*^ and **w**_*j*_^*k*^ representing the target wave functions and PS output wave functions, respectively. Again, following analogy to DVR schemes, the set of PS-predicted wave functions are obtained as the *n*_*e*_ eigenfunctions (with lowest corresponding eigenvalues) of the output workspace matrix **M**.

A final important point concerns the number of grid points used to represent each target problem used in evaluation of eqs 6 and 7. In our initial PS studies, we generated a set of target problems on uniform grids **x** with a *fixed* number of grid points; further analysis demonstrated that, although successful in reproducing target wave functions for the chosen grid size, the resulting PS-generated codes were not transferable to other grid sizes. To combat this, we demonstrated that one can instead generate target problems with a range of different grid sizes, and use these targets as the basis of the cost functions in eqs 6 and 7. Here, because PS optimization is driven toward reproducing the target eigenfunctions for systems with a range of grid sizes, the resulting algorithms are much more generally applicable across different grids. In the simulations reported here, we consider code optimization for both cases: using target problems with either a range of grid sizes (offering transferability) or fixed grids (offering potential to generate optimized codes for each particular grid size).

Algorithm optimization proceeds by using SA to minimize *F* (eq 6 or 7), starting at some initial effective temperature *T*_init_. During a series of *N*_iter_ iterations, the current integer sequence defining a given code is updated by randomly changing a small number (typically between one and three) of integers in the code sequence (corresponding to changing input or internal functions). The new algorithm is accepted or rejected based on the usual Metropolis criterion, where the probability of acceptance of the proposed change is given by
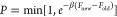
where β is the current effective temperature , *T*, and *F*_new_ and *F*_old_ are, respectively, the cost function values for the new and old code sequences. During the SA iterations, the effective temperature is linearly decreased such that the temperature at iteration *j* is given by
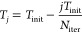
At the end of *N*_iter_ iterations, the resulting algorithm can be further tested by assessing its performance in a set of independently generated random PESs (which were not included during optimization).

Our PS system described above is implemented in a simple standalone python code, using numpy to perform function evaluations where appropriate.^45^ The set of input, output, and internal functions are encapsulated in a function library module that can be readily modified and adapted to the problem at hand. On completion of the SA optimization, the result is an algorithm given as a sequence of *N* integers defining the input workspace vector/matrix definitions, the *N* – 2 internal functions, and the output function (which is, in the current article, simply fixed as an eigenvector calculation). Given that each integer corresponds to a specific function defined in the PS function library, it is straightforward to extract code which implements a given algorithm as a simple sequence of python instructions. Furthermore, because each instruction corresponds to a well-defined mathematical operation, it is also simple to translate output algorithms into working equations giving the elements of the workspace matrix **M**; where appropriate equations defining workspace matrices are given in section 3.

#### 2.3.1 Target Data Generation

The stochastic optimization PS strategy described above requires target data in order to assess algorithm performance using eq 6 or eq 7; specifically, we require *M* input/output examples comprising the PES evaluated on the coordinate grid (as input) and the corresponding numerically exact target wave functions on the coordinate grid (as expected outputs).

In all calculations considered below, target wave functions are generated using the Colbert–Miller DVR method (CM-DVR), a well-known DVR with particularly simple Hamiltonian matrix elements.^34^ The CM-DVR scheme uses a uniform coordinate grid with *n*_*g*_ grid points; in our calculations, the coordinate-grid range is [−5,+5], the grid length is *L* = 10. The CM-DVR has Hamiltonian matrix elements given by
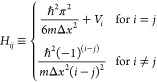
8where *V*_*i*_ is the PES value at grid point *i* and Δ*x* is the grid spacing. For the purposes of later discussion, note that, while the Hamiltonian matrix elements are straightforward to calculate, the resulting Hamiltonian matrix is reasonably dense, with off-diagonal elements decaying to zero as 1/(*i* – *j*)^2^.

Following previous work, we generate target PESs of the form
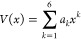
9The coefficients **a** are randomly generated by sampling uniformly in the range [−5,+5]. We ensure that the PES is bound by checking the value of the PES at the upper and lower limits of the coordinate grid, demanding that these values are both greater than *V*(*x*) = 5.0. The requirement of using bound PESs reflects our interest in using DVR-type methods and PS-generated algorithms to study the quantum vibrational properties of typical molecular systems.

After a choice number of grid points *n*_*g*_, generation of random bound-polynomial PESs using eq 9 and diagonalization of the resulting Hamiltonian matrix in eq 8 then gives the target outputs used in eqs 6 and 7. The advantage of the data generation scheme described here is its simplicity; generating new PESs for optimization and independent code-testing is straightforward, and the numerically converged answers can be readily obtained. Finally, we emphasize that, during PS simulations, the target data generated by CM-DVR uses a large grid with *n*_*g*_ = 151 grid points; in other words, the target CM-DVR data represents well-converged results.

### 2.4 New Ideas To Improve PS

Having described our initial PS strategy and its connection to traditional DVR methods, we now turn to focusing on the new aspects of this Article. First, as noted above, our initial implementation of PS focused on seeking algorithms that could reproduce the first few eigenstates of arbitrary bound 1-D PESs. Of course, in addition to eigenstates, a second key required output in useful quantum-chemical calculation is the corresponding energy eigenvalues; as such, in this Article, we focus on methods that provide approximations to both the eigenstates and the corresponding energies. Second, we discuss how one can use PS to generate algorithms that only employ highly sparse (i.e., tridiagonal) matrices, offering significant advantages from a computational-efficiency perspective that cannot be easily obtained in standard DVR schemes; this is demonstrated in applications to few-dimensional systems in section 3.4.1.

#### 2.4.1 Energy Prediction

Generally, for matrix-based schemes for solution of eq 1, there are two routes to calculating the energy eigenvalue for a given allowed eigenstate. First, one can either calculate the energy eigenvalues by diagonalization of the Hamiltonian matrix, as in the CM-DVR scheme. A second, more circuitous route, is to calculate the energy through evaluation of the energy expectation value, given in the 1-D case as
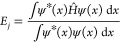
10In principle, evaluation of energies by these two routes should give the same value; however, this assumes that the matrix that is being diagonalized to give the eigenfunctions is an accurate approximation to the true Hamiltonian matrix of the system. In the case of the CM-DVR method (and any other DVR method), this is true by construction, such that both energy evaluation routes effectively give the same result.

However, we must recognize that our PS scheme does not necessarily generate accurate representations of the full Hamiltonian matrix; by their nature, the workspace matrices **M** generated by our PS strategy have been tuned to reproduce selected *eigenfunctions*, without any regard to the corresponding eigenvalues. As such, in the PS scheme reported previously, there is no guarantee that the two different energy evaluation methods would give the same result.

To explore this point, we consider here two modification of the cost function in our PS scheme to enable accurate prediction of *both* eigenfunctions *and* energies; these two methods are as follows:

(1) **Method E1:** The cost function *F* used during SA is given by the root-mean-square fractional error (RMSE) between the energy expectation values (eq 10) calculated for the eigenfunctions generated by PS and the target energy eigenvalues given by CM-DVR:
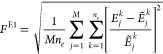
11Here, *Ẽ*_*j*_^*k*^ is the target energy eigenvalue (given by CM-DVR) for the *k*th eigenstate of the *j*th target PES, and *E*_*j*_^*k*^ is the corresponding energy predicted as the expectation value of the Hamiltonian operator; we consider the first *n*_*e*_ eigenvalues for each of the *M* target problems. Note that, because the energy expectation values are evaluated from the wave functions predicted by PS, this cost function implicitly requires both eigenfunctions and eigenvalues to be accurate in order to reach small values of *F*^E1^.

(2) **Method E2**: Here, the cost function used during SA optimization is a composite function based on agreement between both the target and PS wave functions, as well as the energy eigenvalues given by CM-DVR and by diagonalization of the workspace matrix **M**:
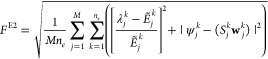
12Here, λ_*j*_^*k*^ is the *k*th lowest eigenvalue of the output workspace matrix **M**, and **w**_*j*_^*k*^ is the corresponding eigenvector. In passing, we note that the magnitudes of the two different components in *F*^E2^ are expected to be roughly similar, particularly given the fact that the eigenvectors **w**_*j*_^*k*^ are normalized on output from a trial algorithm before the cost functions are evaluated.

As a further comment, we note that a third alternative approach, using eq 11 but replacing the calculated energy expectation values with the eigenvalues λ_*j*_^*k*^ is also a possibility; however, in practice, we have found that this approach results in eigenfunctions which are effectively nonsensical, as discussed below. On a practical note, calculation of eq 12 is somewhat complicated by the fact that the PS target problems have different grid-sizes yet the target eigenfunctions provided by converged CM-DVR calculations will necessarily use larger grids. To address this in the calculation of eq 12, we compare each element in the output vector **w** to the *closest* grid point in the target wave function ψ; given the fine grid spacing in the numerically exact CM-DVR results, the error introduced here will be small.

#### 2.4.2 Seeking Sparse Eigenproblems

The second modification of our PS strategy that will be studied here, and perhaps a main outcome of this Article, is the investigation of PS as a tool to develop algorithms for solving eq 1 using matrices that have a predetermined structure that offers computational benefits.

As noted above, the calculation of the Hamiltonian matrix in DVR methods is often quite straightforward.^35^ Unfortunately, in typical direct-product grid-based methods, the number of matrix elements increases rapidly as the number of degrees of freedom increases; as noted above, assuming an average number of *n̅* grid points along each degree of freedom, the direct-product nature of the coordinate grid and wave function representation means that the number of grid points required in an *f*-dimensional system is *n̅*^*f*^. Similarly, this means that the number of elements in the full Hamiltonian matrix is proportional to *n̅*^2*f*^.

The impacts of this rapidly increasing number of grid points (an example of the “curse of dimensionality”) are clear. Multidimensional DVR calculations can require very large numbers of energy evaluations, which can be particularly demanding if one is using high-accuracy ab initio electronic structure calculations. Furthermore, the increasing size of the Hamiltonian matrix can place significant demands on computational memory and storage for multidimensional calculations, such that eigenfunction evaluation can become prohibitively expensive.

As such, there has been a large amount of work focused on reducing the computational effort associated with DVR calculations.^39−41,46^ A primary interest has been the development of efficient pruning methods, which seek to remove those grid points in the full direct-product grid, which are predicted to have essentially zero wave function amplitude for a given PES; for example, research by Carrington and co-workers has clearly demonstrated how pruned basis sets can be developed to significantly reduce the computational cost of DVR calculations.^47,48^ A complementary approach is to seek to reduce the number of grid points required in each DOF, for example by seeking to optimize the choice of underlying DVR basis set to best match the nature of the problem at hand; this is exemplified by the potential-optimized DVR methodology,^41^ as well as developments aimed at using nonproduct coordinate grids.^49^ Finally, we note another alternative to reducing computational effort, namely, the use of ML strategies to generate global PESs using a reduced number of ab initio PES evaluations. In our own recent work,^19,50−56^ we have shown how kernel ridge regression (KRR) can dramatically reduce the number of ab initio PES evaluations required to generate an accurate global PES, particularly if the requisite kernel functions are appropriately chosen. For example, in a recent study of full-dimensional (6-D) nonadiabatic dynamics in thioformaldehyde, ∼300 PES evaluations were required to generate a KRR PES,^54^ while just over 2700 PES evaluations were required when performing 12-mode/2-state nonadiabatic simulations of pyrazine;^53^ in both cases, the required number of PES evaluations is far fewer than the number of DVR grid-points used in modeling the underlying dynamics via multiconfiguration time-dependent Hartree (MCTDH) method.

In addition to the measures outlined above, a further approach to reducing computational effort is to seek methods for solving eq 1 that retain the simplicity of DVR schemes but have computationally beneficial algorithm structure. Specifically, it is well-known that *sparse* matrices (dominated by a large number of elements that are essentially zero) offer significant computational advantages when one is handling matrix and matrix-vector manipulations;^42,57^ for example, sparse matrices can exploit efficient indexing schemes to minimize storage requirements, while efficient iterative schemes for eigenproblem solution readily benefit from sparsity in the associated matrix manipulations. In such methods, the computational time for matrix-vector multiplications becomes roughly proportional to the number of nonzero matrix elements in a sparse matrix; in other words, minimizing the number of nonzero elements (i.e., maximizing sparsity) offers a route to computational benefits.

In the “best-case” scenario, it seems that a method for solving eq 1 that employs a *tridiagonal* Hamiltonian (or workspace) matrix could be considered optimal; it is hard to imagine a simpler or more compact matrix structure that could give equally accurate eigenvalues and eigenfunctions. However, in standard DVR schemes, there is little opportunity to derive accurate methods that result in exclusively tridiagonal Hamiltonian matrices; for example, in the CM-DVR method demonstrated in eq 8, it is clear that the resulting Hamiltonian matrix is quite dense. More generally, in alternative DVR schemes using orthogonal polynomial basis sets, while the 1-D position-operator matrix elements might adopt a sparse tridiagonal form, the evaluation of the full Hamiltonian matrix in the DVR basis requires matrix multiplications by matrices that are not necessarily sparse (and, hence, result in a nonsparse Hamiltonian matrix).^35^

However, because our PS strategy is not tied to an underlying basis or DVR transformation, imposing target properties on the final workspace matrix **M** is quite straightforward. Specifically, in the results considered in section 3.4 below, we use PS to generate algorithms that give accurate eigenfunctions and eigenvalues, but which *only* use tridiagonal matrices in solving the associated 1-D eigenproblem. In the context of our PS approach, this can be simply achieved by setting all elements of the output workspace matrix **M** to zero, except for those on the diagonal (*M*_*ii*_) or the diagonal-adjacent bands (*M*_*i*,*i*±1_). After enforcing this tridiagonal structure, the predicted wave functions of the target PES are obtained by diagonalizing **M**, with the energy eigenvalues obtained from either diagonalization of the matrix or as expectation values. The resulting PS-generated codes are optimized in exactly the same manner as described above; the only difference is the imposition of tridiagonal structure in **M** before the final evaluation of algorithm performance. We note that this same scheme can, of course, be used to impose other (e.g., banded) matrix structures; we consider tridiagonal matrices exclusively here because they represent the best-possible case, in terms of matrix sparsity, and, hence, result in excellent matrix sparsity for multidimensional problems too. Finally, we emphasize that the workspace matrix **M** will be tridiagonal in the case of 1-D problems; for multidimensional problems, the structures of eqs 4 and 5 imply that the resulting workspace matrix will no longer by tridiagonal, but, below, we clearly demonstrate that imposing tridiagonal structure on the 1-D subproblems still has large sparsity benefits for multidimensional problems.

In summary, building on our initial PS approach to generate algorithms for wave function prediction on uniform grids, this Article extends our strategy to (i) investigate two different schemes for energy evaluation in PS, and (ii) employ PS to identify new grid-based algorithms for solving eq 1 using tridiagonal (and highly sparse) matrices. The results of these investigations are discussed in the following section.

## 3 Simulations, Results, and Discussion

In this section, we consider the impact of the two updates of our initial PS strategy described in section 2.4. We initially focus on using 1-D PESs as target input/output data for our stochastic PS optimization strategy. As noted above, there is an analogy between grid-based DVR methods and the grid-based algorithms generated by our PS approach; as a result, 1-D DVR-like algorithms generated by PS can be readily extended to multidimensional systems (e.g., eqs 4 and 5), just like standard 1-D DVR methods can. So, although we focus on using 1-D examples as target data for PS optimization, the resulting codes can be generalized to multidimensional systems; in fact, we show below that PS-generated DVR-type algorithms for multidimensional problems can have advantages over traditional DVR algorithms.

### 3.1 Simulation Details

Here, we summarize the general PS simulation conditions used for all calculations below; specific further details are given in the relevant sections.

In all PS optimizations, a total of *M* = 20 target input/output examples were used, with the mass of the corresponding degree of freedom assumed to be *m* = 1 in all cases. These PES examples were generated as described in section 2.3.1. A total of 100 independent PS optimizations were performed for each of the different PS simulation conditions outlined below, with each using a different set of target input/output data; for example, for each cost function evaluation scheme (e.g., method E1 or E2) and code-size considered (e.g., *N* = 15, 20, and 25), we performed 100 independent optimization simulations. Furthermore, all calculations described below used the first *n*_*e*_ = 3 eigenstates as targets, with the PS simulations aiming to reproduce the wave functions *and* energies of these states. We note that this number of target states is somewhat arbitrary, and could be increased; however, as in all grid-based DVR-type methods, the finite grid places an implicit limit on the accuracy with which higher eigenfunctions can be represented. In other words, although we anticipate that larger values of *n*_*e*_ could be targeted, the tradeoff is the requirement of larger grid sizes; the problem setup described here is suitable to evaluating the first few vibrational wave functions and energies for bound molecular systems. Furthermore, given the analogy between the PS-generated codes identified below and DVR schemes, we show later that our PS schemes can in fact accurately reproduce the wave functions and energies of higher-energy states than just the *n*_*e*_ = 3 states used in training.

In each SA optimization, the initial temperature was *T*_init_ = 5 × 10^3^ K, and a total of 5 × 10^3^ code updates were attempted. At the end of each simulation, our python code lists the set of instructions for the best discovered algorithm; where required, these code instructions were translated into working equations by hand. We note that symbolic computation^58^ could alternatively be used to automatically simplify working equations, but that approach is not pursued here.

When using method E1, we evaluate the integrals required in eq 10 using standard numerical techniques. For the PES component, where the wave function ψ(*x*) and the PES *V*(*x*) are known at a set of grid points **x**, we use the standard trapezoid rule to evaluate the integral (using the numpy library^45^). In the case of the KE contribution, we first use KRR to generate a continuous representation of the PS-predicted wave function (thereby enabling approximation of the required second derivative); here, the wave function is written as a linear combination of Gaussian kernel functions,
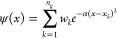
13where the weights *w*_*i*_ are obtained by solving the simultaneous equations requiring the wave function to be correctly reproduced at each grid point, namely,
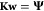
14Here, *K*_*ij*_ = *e*^–α(*x*_*i*_–*x*_*j*_)^2^^, **w** is the vector of *n*_*g*_ unknown weights, and Ψ is a vector containing the PS-predicted wave function on the grid. In the PS simulations reported below (many of which use different grid sizes *n*_*g*_), in order to enable consistent representation of ψ(*x*), the width parameter α is chosen so that the Gaussian kernel functions have a constant value at an adjacent grid-point, independent of grid size. So, we require
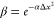
where β is a target Gaussian kernel value at adjacent grid points (chosen here to be β = 0.5), and Δ*x* is the grid spacing. Rearranging gives
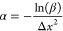
With a continuous representation of ψ(*x*) in hand, the second derivatives can be evaluated as
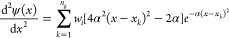
15which then enables evaluation of the KE contribution to eq 10 by numerical integration. We note that the evaluation of energy expectation values in this way inevitably introduces numerical errors that must be factored in when judging algorithm performance; however, the results below demonstrate that this method enables accurate energy evaluation across the grid sizes considered here, as noted below.

A further methodological point involves the identification of “high-performing” codes. Our primary goal is to determine whether PS can be used to accurately predict wave functions and energies for bound PESs, and our chosen accuracy criteria reflect this goal. Specifically, in the following, we will use a cost function value (eqs 11 and 12) of 2 × 10^–2^ (for the training data) to represent a “good” algorithm, which should be investigated further; in the case of method E1, we note that this target implies an average error of <2% in calculated energy expectation values (relative to numerically exact DVR calculations), although the combined nature of the cost function in method E2 means this interpretation is less straightforward (as discussed in more detail in section 3.2).

For those algorithms that are deemed to be “high-performing” based on the cost function criteria for the target example data, we subsequently perform independent tests of accuracy and convergence with grid size. To do so, we generate a further independent test set of 500 1-D PES examples, calculate the numerically exact (i.e., large grid-size) CM-DVR energies, and compare these to the predictions made by a given PS-generated code.

### 3.2 Comparison of Energy Evaluation Schemes

We first compare the utility of different cost functions, E1 and E2. As noted above, method E1 uses PS to predict target wave functions that are subsequently used to evaluate energy expectation values by numerical integration, whereas method E2 takes the energy eigenvalue predictions from the PS-generated workspace matrix **M** (as in typical DVR schemes).

We note that, in these simulations, there are no matrix-structure requirements, such as tridiagonal or banded structure, placed on the workspace matrix **M**. Furthermore, these simulations used a set of PESs and coordinate grids with randomly generated (odd) numbers of grid points *n*_*g*_ ∈ [15, 91] as target data; while the requirement of using an odd number of grid points is not essential, it satisfies the condition of always placing a grid point at the center of the coordinate range [−5,+5], providing a center-of-symmetry in all coordinate grids (noting that this does *not* imply that the PESs used are symmetric, as demonstrated below).

**Figure 2:**
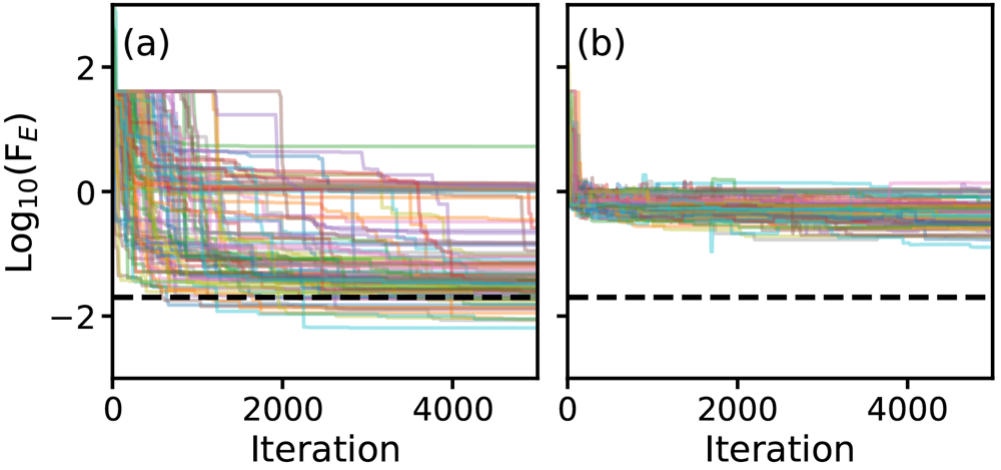
Root-mean fractional errors in calculated energy eigenvalues (eq 11) as a function of SA iteration from 100 independent PS simulations using (a) method E1 or (b) method E2. The horizontal dashed line represents the target fractional error of 2 × 10^–2^; note the logarithmic plot on the *y*-axis.

Figure 2 shows the progression of the root-mean-square (RMS) fractional errors in energy eigenvalues during 100 independent PS simulations for code sizes *N* = 20; Figure 2a shows the results of simulations performed using method E1, whereas Figure 2b shows results using method E2. In the case of method E2 (Figure 2b), in which the cost function is a composite of an energy component and a wave function component, we only show the energy contribution; in other words, although the results from Figure 2b were obtained using the cost function in eq 12, the plotted data only show the energetic contribution in eq 11 to enable closer comparison of the two different approaches.

Assuming a target RMS fractional error of 2 × 10^–2^ (i.e., percentage error of 2%), we find that a total of 19 out of 100 simulations using method E1 successfully identify a high-performing algorithm; in contrast, none of the algorithms determined using method E2 were able to identify codes with RMS fractional errors of <2 × 10^–2^. We note that increasing the number of allowed code instructions from *N* = 20 to *N* = 25 does not improve the success of method E2.

The results of Figure 2 demonstrate that method E1 (using wave function expectation values to predict energies) enables discovery of accurate algorithms using PS, but method E2 (using matrix eigenvalues as energy predictions) does not. In seeking to explain this result, the first question is whether or not the *wave functions* predicted by method E2 are accurate; because the cost function in E2 is a composite of both energy and wave function contributions, it is worth investigating how the codes discovered by PS using method E2 perform for these separate contributions.

**Figure 3:**
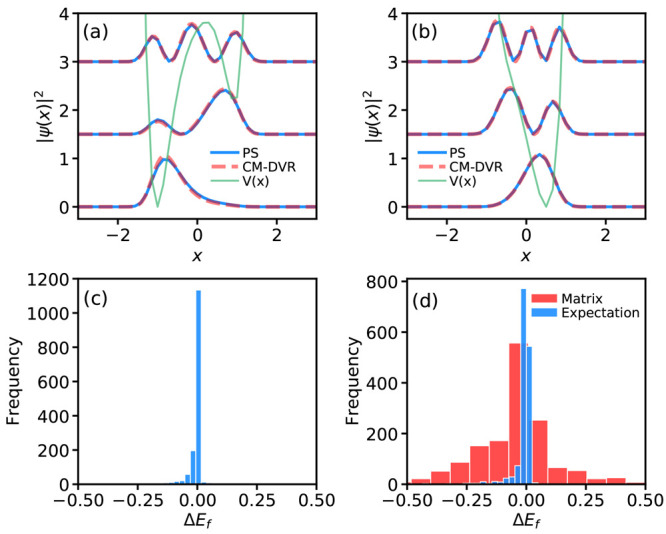
Comparison of performance of algorithms *C*_20_^Full^(E1; *A*) and *C*_20_^Full^(E2; *A*). (a) Probability distributions |ψ(*x*) |^2^ for the first three eigenstates of a randomly generated PES (green), calculated by CM-DVR (red dashed) and algorithm *C*_20_^Full^(E1; A) (blue solid). (b) Same as panel (a), but PS results were calculated using algorithm *C*_20_^Full^(E2; A). (c) Histogram of fractional errors Δ*E*_*f*_ for first *n*_*e*_ = 3 eigenstates for 500 randomly generated PESs, with energies calculated as expectation values of eigenstates given by algorithm *C*_20_^Full^(E1; A). (d) Histogram of fractional errors Δ*E*_*f*_ for first *n*_*e*_ = 3 eigenstates for 500 randomly generated PESs, as given by algorithm *C*_20_^Full^(E2; *A*), with energies predicted by either matrix eigenvalues (red, labeled “Matrix”) or as expectation values of the Hamiltonian operator for the corresponding predicted wave functions (blue, labeled “Expectation”).

Figure 3a shows the first three wave functions predicted for a randomly generated PES by CM-DVR (with a large grid size of *n*_*g*_ = 151 grid points) and by the best-performing algorithm obtained by PS using method E1 (and grid size *n*_*g*_ = 71, chosen at random for this PES). This “best” algorithm had a RMS fractional error of 8 × 10^–3^ for the PS target data; the data shown in Figure 3a are for an independent random PES, which was not included during PS optimization. Following our previous work, we refer to the best-performing algorithm as *C*_20_^Full^(E1; A); here, the subscript identifies the number of code instructions (*N* = 20 in this case), the parentheses identifies the cost function used during PS optimization and assigns a letter to identify different algorithms, and the superscript “Full” indicates that the full matrix could be modified during the PS optimization (in contrast to the tridiagonal matrix structure considered below in section 3.4). We note that the algorithm *C*_20_^Full^(E1; A) is discussed in more detail in Section 3.3; in this section, we focus on comparing methods E1 and E2.

It is immediately clear from Figure 3a (as well as by comparison of numerically exact and PS-generated wave functions for other PESs) that the reproduction of wave functions using *C*_20_^Full^(E1; A) is excellent; we note that the randomly generated PES in this case exhibits two minima, and is not just a simple parabola, but the wave functions obtained by *C*_20_^Full^(E1; A) agree very well with those from CM-DVR. Figure 3b then shows wave functions obtained for a random PES by the best algorithm obtained using E2 as the cost function (i.e., *C*_20_^Full^(E2; *A*)). Again, the agreement with the wave functions obtained by CM-DVR is very good, and is found to be similarly good for a broad range of random PESs. In other words, it is clear that both methods E1 and E2 can deliver algorithms which yield very good reproduction of *wave functions*.

We now consider the *energy* predictions from the best algorithms, *C*_20_^Full^(E1; A) and *C*_20_^Full^(E2; A), as shown in Figures 3c and 3d. In Figure 3c, we show the distribution of fractional errors,
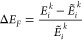
calculated for 500 randomly generated PESs. Here, *E*_*i*_^*k*^ is the predicted energy of the *k*th state for the *i*th random PES, and *Ẽ*_*i*_^*k*^ is the corresponding numerically exact value obtained from CM-DVR. For algorithm *C*_20_^Full^(E1; A), the RMS fractional error after optimization was 8 × 10^–3^, and this is reflected in the very narrow distribution of fractional errors observed in Figure 3c. Figure 3d also shows the errors Δ*E*_F_, but for predictions made by algorithm *C*_20_^Full^(E2; A). In this case, the Δ*E*_F_ values calculated for the matrix energy eigenvalues are shown, as are the Δ*E*_F_ calculated as energy expectation values using the PS output wave functions; as a reminder, method E2 optimizes a cost function that includes energy eigenvalue predictions obtained from the workspace matrix **M**. Even though the energies obtained from **M** served as optimization targets, it is clear that algorithm *C*_20_^Full^(E2; A) does not perform well in predicting energies, as highlighted by the very broad distribution of fractional errors for matrix eigenvalues in Figure 3d. In contrast, the distribution in fractional errors obtained by evaluating energies as expectation values is much narrower, and comparable to that shown in Figure 3c. As such, the conclusion of the analysis in Figure 3 is that both methods E1 and E2 provide accurate routes to generating algorithms that accurately predict *wave functions*, but the prediction of accurate energies from matrix eigenvalues is much more challenging; in contrast, given that both methods E1 and E2 predict accurate wave functions, evaluating energies as expectation values is more accurate.

As the final point in this section, we comment on why prediction of energies using matrix eigenvalues appears to be more challenging than prediction of the corresponding eigenfunctions. This observation can be explained by considering the degeneracy of problems associated with finding either eigenvalues or eigenvectors. As is well-known, the eigenvalues of a square matrix **A** are given as the roots of the characteristic polynomial,
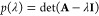
where λ is an unknown eigenvalue and **I** is the identity matrix. The eigenvalues of a given matrix are invariant under the action of similarity transformations of the form **A** → **C**^–1^**AC**, where **C** is a square matrix; in the context of PS, this means that any square matrix which is related to the true Hamiltonian matrix via a similarity transformation will have the same eigenvalue spectrum, and so could provide an optimal solution of eq 11 or 12. In other words, we anticipate that there should be a number of different PS-generated algorithms which give good reproduction of the energy eigenvalues for a given PES when predicted from the workspace matrix **M**; however, it is clear that locating these matrices using our current PS system is extremely challenging.

Now consider the problem of identifying matrices which reproduce the *eigenfunctions***u** of a matrix **A**; we assume that the matrix has eigenvalues λ. For a broad class of matrix functions *f*(**A**) (e.g., polynomials and functions which can be expressed as Taylor expansions), the eigenfunctions of **A** are equally eigenfunctions of *f*(**A**), with eigenvalues *f*(λ). For example, if **Au** = λ**u**, then
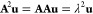
As a second example,
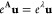
which can be confirmed by considering the Taylor expansion of the exponential function. The key point is that there is a *very* large number of matrices that all share common eigenvectors but have completely different eigenvalue spectra; for example, in the two examples given above, the eigenvectors **u** are common to both **A**^2^ and *e*^**A**^, but the eigenvalues (λ^2^ and *e*^λ^) are completely different.

What does this suggest for methods E1 and E2? The implication of these eigenproblem properties are that there are a very large number of matrices (or matrix functions) which have very similar eigenvectors to those given by a true matrix representation of the Hamiltonian operator, but there are only a (relatively) small number of matrices that have the same eigenvalue spectrum as the Hamiltonian matrix (i.e., similarity transformations). In an additional complication, we note that, although similarity-transformed matrices exhibit the same eigenvalue spectrum as the original matrix, the same is not necessarily true with regard to the eigenvectors. In a stochastic optimization procedure, such as the SA method used in our PS strategy, it is therefore much “easier” to identify a workspace matrix **M** which reproduces the correct eigenvectors for a given PES (as employed in method E1, and our previous work), whereas identifying a matrix **M** which yields both correct eigenvectors *and* eigenvalue spectrum appears to be a much more challenging prospect; in the latter case, using stochastic optimization to find matrices with correct eigenvalue spectrum appears akin to “finding a needle in a haystack”. This could explain why method E2 is not as successful as method E1 in delivering accurate eigenvalue predictions; in short, E1 only needs to reproduce the eigenvectors (and there are many ways to achieve this), whereas E2 needs to reproduce both eigenvectors and eigenvalues (and it seems more challenging to achieve this, at least given the current PS setup). Furthermore, we note that the same underlying reason most likely explains why the alternative method of using eq 11, but with energy eigenvalues taken from matrix diagonalization, also proves unsuccessful. Again, accurate reproduction of the correct energy eigenvalues by matrix diagonalization requires that **M** is an accurate representation of the true Hamiltonian operator evaluated in some basis representation; with the current PS setup, this appears to be difficult to achieve.

### 3.3 High-Performing Full-Matrix Codes

At this point, it is interesting to investigate the performance of full-matrix codes for uniform grids which enable accurate reproduction of both wave functions *and* energies using method E1 (which is used exclusively hereafter). Here, we performed PS simulations using three different code sizes, *N* = 15, 20, and 25, with all simulations performed as described in section 3; specifically, for each code size, we performed 100 independent PS optimizations. Assuming a target accuracy of *F*_E1_ < 2 × 10^–2^, we find that the success rate is ∼20%, regardless of code size *N*; however, rather than presenting full details of the ∼60 algorithms that satisfy this performance criterion, we simply focus on the best algorithm of each code size.

Based on the instruction sets generated for each algorithm, it is straightforward to write down the corresponding output matrix approximation **M** which is generated by each algorithm; these are shown in Table 1. As expected, these matrix approximations are implicitly “DVR-like”, in the sense that the PES values at the *n*_*g*_ grid points appear in the diagonal elements, whereas the off-diagonal elements are generally decaying functions depending on (*x*_*i*_ – *x*_*j*_). Beyond being able to write down the working equations of these matrix approximations, seeking additional rationalization of these schemes is challenging. However, based on the discussion in section 3.2, it is perhaps now clear why; in particular, we have highlighted above that a large number of arbitrary matrix functions can have the same eigenvectors as the target Hamiltonian matrix, so it is not surprising that the resulting matrix approximations in Table 1 are somewhat opaque.

**Table 1:** Derived Equations for Output Workspace Matrices **M** for Best-Performing Algorithms with Code Sizes *N* = 15, 20, and 25^a^

code size *N*	code label	output matrix elements, *M*_*ij*_
15	*C*_15_^Full^(E1; A)	
20	*C*_20_^Full^(E1; A)	
25	*C*_25_^Full^(E1; *A*)	

a Final predicted wavefunctions are obtained as the eigenvectors of **M**; we use the numpy routine eigsh to obtain eigenvectors,^45^ which assumes that the input matrix is symmetric and the lower-triangular portion is input.

**Figure 4:**
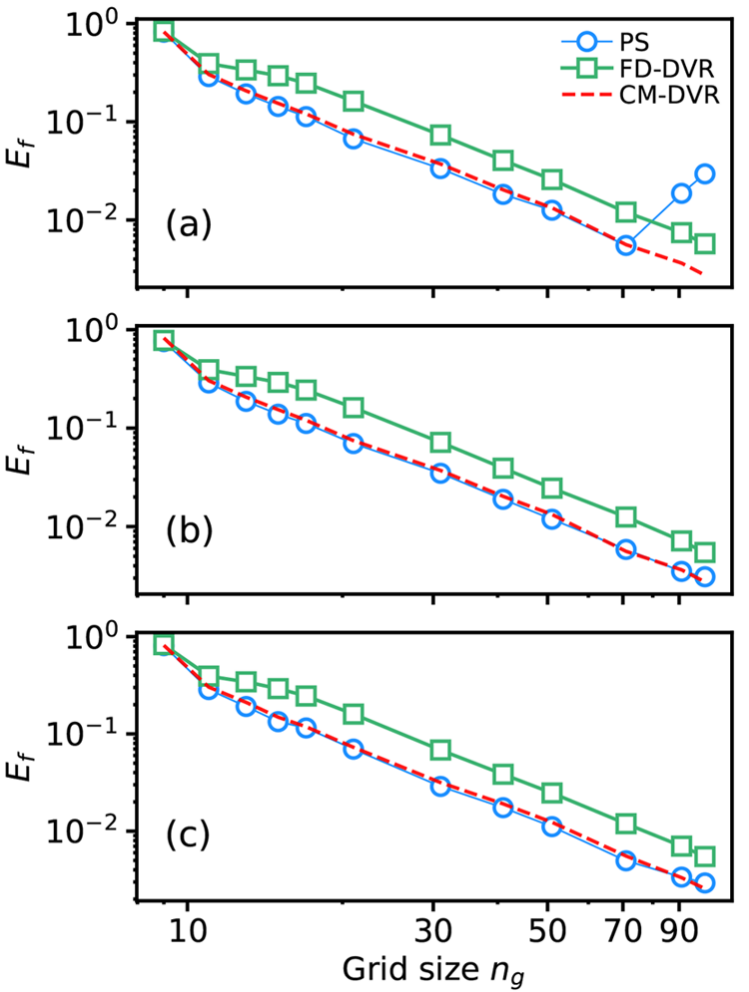
Grid-size convergence of root-mean-square (RMS) fractional error (relative to CM-DVR calculations with *n*_*g*_ = 151) of DVR and PS-generated algorithms (a) *C*_15_^Full^(E1; A), (b) *C*_20_^Full^(E1; A), and (c) *C*_25_^Full^(E1; A). In each panel, we show the results of calculations for the PS algorithms (blue circles), the finite-difference DVR of eq 16 (green squares), and the Colbert–Miller DVR of eq 8 (red dashed). At each grid size, the RMS fractional errors are calculated for each method for the first *n*_*e*_ = 3 eigenstates for 500 randomly generated polynomial PESs; error bars are typically much smaller than the symbol size, and are not shown, for the sake of clarity.

What is clear, however, is that the algorithms shown in Table 1 exhibit very good accuracy in reproducing both target wave functions and energy values; this is highlighted in Figure 4. In particular, Figure 4 shows the convergence of the RMS fractional errors in the *n*_*e*_ = 3 eigenvalues for 500 randomly generated PESs, as obtained by (i) the algorithms from Table 1 (with energies calculated explicitly as expectation values), (ii) CM-DVR calculations with a large grid size, and (iii) a simpler DVR scheme with KE matrix elements obtained by central finite differences, resulting in the following Hamiltonian matrix:
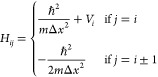
16In all cases, the RMS fractional errors are calculated relative to CM-DVR calculations performed using *n*_*g*_ = 151 grid points. Here, we define *E*_*f*_ as the RMS fractional error in the test-set calculations; this is calculated using eq 11.

The results of Figure 4 demonstrate that the PS-generated algorithms of Table 1 can exhibit slightly better convergence in RMS fractional errors when compared to the CM-DVR algorithm. This is a useful achievement in demonstrating the potential of PS; a computer-discovered algorithm can solve eq 1 at a better level of accuracy than standard “human-derived” algorithms, even when inevitable inaccuracies due to numerical integration are taken into account. Of course, the benefit in accuracy is, as might be expected, quite small; typically, we find that the RMS fractional errors given by the algorithms in Table 1 are ∼0.5% smaller than those given by CM-DVR (although, given the accuracy of CM-DVR, this usually represents an improvement of up to 10% on the RMS fractional errors given by CM-DVR). Furthermore, we note that the finite-difference DVR method performs much worse than either CM-DVR (as expected) or PS-generated codes.

Another important observation relates to the overall convergence behavior of the PS-generated algorithms. Because the algorithms in Table 1 were obtained using target input/output data with a range of grid sizes, we anticipate that the performance of these methods should be maintained across the training grid-size range of *n*_*g*_ ∈ [15,91]; this is generally found to be the case for code sizes *N* = 20 and *N* = 25, with these algorithms offering slightly lower RMS fractional errors than CM-DVR in this grid range. In the case of the best *N* = 20 and *N* = 25 codes, the PS-generated codes demonstrate essentially the same convergence behavior with grid size as the CM-DVR method. In the case of the *N* = 15 code, we find that the convergence follows that of CM-DVR, except for the largest grid sizes; given that the random grid sizes for the *N* = 15 code were generated in the same manner (and broadly cover the same range) as those generated for *N* = 20 and *N* = 25, it appears that this divergence at larger grid sizes is an inherent property of this particular algorithm.

As an aside, we note that the best *N* = 15 algorithm (Table 1) is actually an example of a tridiagonal output matrix structure; this has arisen because one of the possible input options is a tridiagonal matrix filled such that *M*_*ij*_ = 1 in all available tridiagonal elements *j* = *i* ± 1, and with all diagonal elements *M*_*ii*_ = 1. As noted above, the large grid-size convergence of this particular algorithm leaves something to be desired; however, in section 3.4, we explicitly generate much larger numbers of tridiagonal matrix algorithms, and show that the best-performing codes in that case exhibit better convergence properties than the *N* = 15 algorithm *C*_15_^Full^(E1; *A*) in Table 1.

Finally, note that one can, of course, use PS to generate algorithms that are optimized to work for a single specific grid size, rather than generalizing across different grids. Results of such simulations are given in the Supporting Information. It is found that the grid-targeted algorithms generally improve on the codes generated for grid ranges (Figure 4), as might be expected; for example, whereas the average RMS fractional errors obtained by the algorithms in Figure 4 are around 0.5%–1.0% smaller than the corresponding CM-DVR results, in the case of grid-specific codes, it is found that the errors are decreased further still, reducing the RMS fractional errors relative to converged CM-DVR by up to ∼9%. Of course, the price paid for this small improvement is the requirement of using different algorithms for different grid sizes, which is not particularly convenient if one is interested in using PS-generated codes in general analysis of quantum molecular vibrational properties.

To summarize, we have shown that PS-generated codes can reduce the errors in predicted wave functions and energies when compared to the CM-DVR method with the same grid size. In the case of PS-generated codes, we have discussed how determination of the correct energy eigenvalues is complicated by the degeneracy of matrix functions with the same eigenfunctions; the required energy eigenvalues can be subsequently evaluated as expectation values, but this is not quite as “neat” as obtaining the energies from solution of the Hamiltonian matrix eigenproblem. That said, given that evaluation of the PES on DVR grids is commonly the time-consuming part of such calculations, numerical evaluation of expectation values is not necessarily expected to be the bottleneck for the PS-based methods generated here (as also discussed below).

### 3.4 Sparse Algorithms Discovered by PS

In the algorithms generated in section 3.3, there was no constraint imposed on the matrix structure. That being said, we did discover a high-performing algorithm [*C*_15_^Full^(E1; A)] which possessed a tridiagonal matrix structure; this was identified by PS because one of the input options comprised a tridiagonal matrix, and the generated code did not include any operations which served to modify this structure. However, as shown in Figure 4, the convergence of *C*_15_^Full^(E1; A) was not very good at large grid sizes.

In this section, we focus on *only* generating algorithms which use a tridiagonal matrix structure in the output matrix **M**; this is achieved by simply zeroing matrix elements *M*_*ij*_ which sit outside the tridiagonal band. Beyond this modification of the output matrix structure, all other aspects of the PS approach were as outlined in sections 2 and 3.1, and employed method E1 during optimization.

PS optimizations were performed to identify tridiagonal matrix algorithms with both *N* = 20 and *N* = 25; the best-performing algorithm was found for *N* = 20, with a RMS fractional error (relative to converged CM-DVR calculations) of 3 × 10^–2^ for an independent test set of 500 randomly generated PESs with random grid sizes *n*_*g*_ ∈ [15, 91]. We emphasize here that our simulations identified ∼10 algorithms, which demonstrated very accurate final optimization-function values of <1 × 10^–2^. Here, we focus on highlighting the energy predictions of just one of these high-performing algorithms; closer analysis suggests that the predicted wave functions of some of the other algorithms are slightly better than the single algorithm studied here, but at the expense of slightly worse energy-predictive performance. We leave the analysis of a broader range of algorithms for future work; the results below are sufficient to demonstrate the PS performance possibilities.

For the best-performing *N* = 20 algorithm, labeled *C*_20_^Tri^(E1; A) to emphasize the imposition of tridiagonal matrix structure during PS, the working equation for the output matrix **M** is
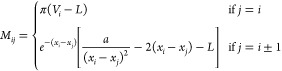
17where *a* = . Wave function predictions are given as the eigenvectors of this matrix, in our case calculated using the numpy function eigh,^45^ which implicitly treats the matrix as symmetric and employs the lower-triangular section as input. As in the case of algorithms derived for the full matrix structure, and in typical DVR algorithms, eq 17 incorporates the PES values on the grid in the diagonal elements; the remaining contributions to diagonal and off-diagonal elements represent contributions from the KE operator (or functions thereof; see section 3.2). In short, the form of this approximation is really no different from those presented in Table 1; the important difference in eq 17 is that only elements in *M*_*ij*_ with *j* = *i* or *j* = *i* ± 1 are nonzero.

**Figure 5:**
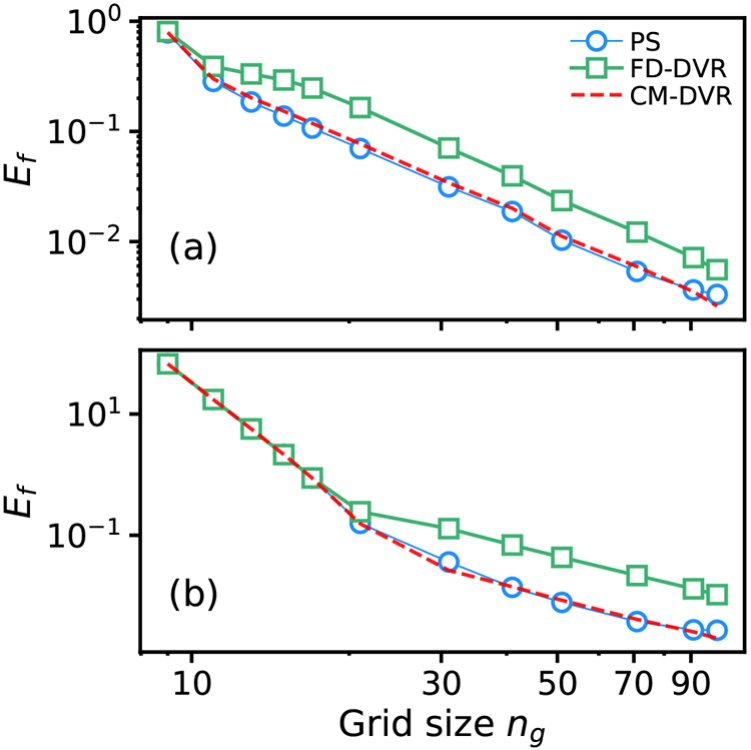
Grid-size convergence of root-mean-square (RMS) fractional error (relative to CM-DVR calculations with *n*_*g*_ = 151) of DVR methods and tridiagonal PS-generated algorithm *C*_20_^Tri^(E1; A) 500 randomly generated PESs. Panel (a) calculates the RMS fractional error for the first *n*_*e*_ = 3 eigenstates, whereas panel (b) uses *n*_*e*_ = 6. We show results from *C*_20_^Tri^(E1; A) (blue circles), the finite-difference scheme of eq 16 (green squares), and the Colbert–Miller DVR (red dashed line).

Further simulations were performed to assess the convergence of *C*_20_^Tri^(E1; A) with different grid sizes; the results are shown in Figure 5a. The results are generally comparable to those obtained using algorithms which generated full, dense matrices (Figure 4); in particular, our PS-generated algorithm typically reduces the RMS fractional error in the energy eigenvalues (relative to converged CM-DVR results) by ∼0.5%–2%. At the largest grid size (*n*_*g*_ = 101), the decrease in RMS error slows somewhat, most likely as a result of the fact that we are using numerical integration to evaluate the energy expectation values (which will inevitably introduces errors which become significant as the magnitude of the RMS errors approaches small values). In addition, the PS optimizations were performed with grid sizes of *n*_*g*_ < 91, so the performance at *n*_*g*_ = 101 might not necessarily be expected to continue to follow the CM-DVR trend.

Figure 5a compares the accuracy of our tridiagonal algorithm *C*_20_^Tri^(E1; A) to another tridiagonal approach based on the simple central finite-difference scheme of eq 16; the difference is significant. Even though PS-generated code *C*_20_^Tri^(E1; A) has the same tridiagonal structure as eq 16, *C*_20_^Tri^(E1; A) demonstrates a far superior convergence of energy values, which has been found to be directly comparable to the CM-DVR method. This clearly demonstrates an advantage of PS, namely, generation of accurate algorithms with computationally beneficial structures (as demonstrated below), which are otherwise difficult to impose.

It is also interesting to highlight Figure 5b, which shows the convergence of energy eigenvalues using *C*_20_^Tri^(E1; A), but for the first *n*_*e*_ = 6 eigenvalues in 500 randomly generated PESs. As a reminder, *C*_20_^Tri^(E1; A) was optimized by PS using a smaller target number of eigenvectors (i.e., *n*_*e*_ = 3). However, Figure 5b demonstrates that the convergence of *C*_20_^Tri^(E1; A) for the first *n*_*e*_ = 6 eigenvalues remains essentially the same as in CM-DVR. We postulate that this performance for higher eigenfunctions is a result of two factors. First, by construction, *C*_20_^Tri^(E1; A) is already a DVR-like approximation to an effective Hamiltonian (as discussed above), with PES values appearing on diagonal elements; the approximation of higher-order eigenfunctions will similarly benefit from this feature. Second, because the “higher” eigenfunctions (*n*_*e*_ > 3) of **M** must be orthogonal to the “lower” eigenvectors (*n*_*e*_ ≤ 3), which are used as optimization targets, this may impose physically sensible features on the higher eigenfunctions and hence improve the accuracy to which they are approximated. Further study of transferability is beyond the scope of this paper, but we aim to investigate this feature in future work.

#### 3.4.1 Sparse Algorithms in Multidimensional Problems

We have shown that PS can be used to develop algorithms satisfying certain target performance criteria (e.g., reproduction of eigenvectors and eigenvalues for 1-D quantum systems) while simultaneously imposing target algorithmic structure on the computer-generated codes. In the case above, we have shown how PS can be used to generate algorithms which require calculation and diagonalization of a tridiagonal matrix (for 1-D problems); this has the advantage of being highly sparse, so that efficient matrix storage and manipulation routines could be used. In contrast, the well-known CM-DVR algorithm (and, in general, other DVR algorithms) exhibit a denser matrix structure, with a larger number of matrix elements taking some numerical value when compared to the tridiagonal case. In this section, we provide a further examination of the sparse algorithm *C*_20_^Tri^(E1; *A*) in the context of multidimensional DVR calculations in order to demonstrate the potential for computational gains using PS-generated codes.

First, we consider the properties of the matrices required by CM-DVR and *C*_20_^Tri^(E1; A) when calculating the eigenvectors for multidimensional systems. As a reminder, for a standard (nonpruned) DVR grid, and assuming that the same number of grid points *n*_*g*_ are used for each dimension for simplicity, the total number of grid points grows as *n*_*g*_^*f*^, where *f* is the number of degrees of freedom in the system; the corresponding size of the required DVR matrices in standard methods is *n*_*g*_^2*f*^. However, when constructing DVR Hamiltonian matrices for multidimensional systems, the effective orthogonal structure of basis functions in different degrees of freedom, as shown in eqs 4 and 5, means that many of these *n*_*g*_^2*f*^ matrix elements are zero, implicitly providing a sparse matrix structure. In the case of DVR-type algorithms built from tridiagonal matrices, this sparsity would be expected to be even more apparent. This sparsity could be important in the case of multidimensional systems, because it can be exploited by sparse matrix storage algorithms and iterative for eigenproblem solution.^35,42,57^ In particular, matrix eigensolution routes based on repeated matrix-vector multiplication operations can benefit greatly from sparse matrix structures; as such, it is interesting to assess the impact of a tridiagonal DVR-type scheme on the efficiency of eigenvector prediction in higher-dimensional systems.

**Figure 6:**
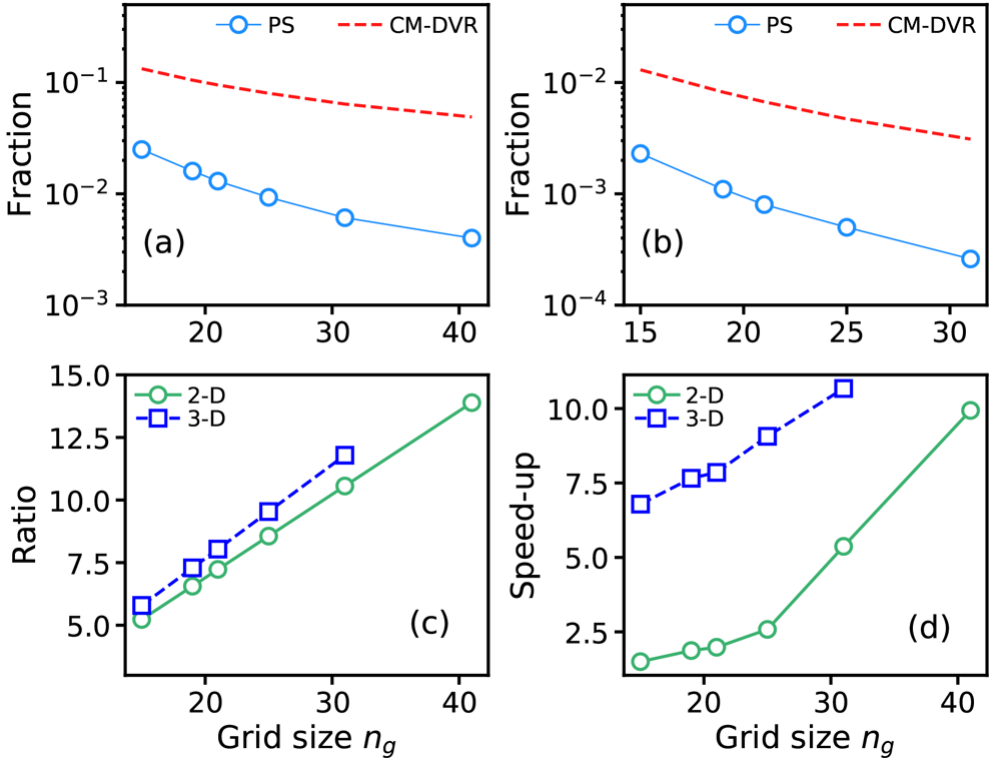
Upper panels show the fraction of nonzero elements in working matrices for tridiagonal methods (e.g., *C*_20_^Tri^(E1; A); blue circles) and the Colbert–Miller DVR (red dashed line) for (a) 2-D and (b) 3-D systems as a function of grid size *n*_*g*_. Panel (c) shows the relative ratios of the number of nonzero matrix elements in 2-D and 3-D systems for *C*_20_^Tri^(E1; A) and CM-DVR, derived from the data in panels (a) and (b). Finally, panel (d) shows the acceleration in eigenproblem solution, given by the relative calculation times for CM-DVR and *C*_20_^Tri^(E1; A) matrix eigensolutions using sparse scipy routines.

To investigate the difference between CM-DVR and the tridiagonal PS-generated code *C*_20_^Tri^(E1; A), Figure 6 highlights properties of matrix eigenvector calculations using these two different approaches. First, Figures 6a and 6b show the fraction of nonzero elements in the final Hamiltonian or workspace matrices for 2-D (Figure 6a) and 3-D (Figure 6b) model PESs with different uniform grid sizes; specifically, the PESs used here were the 2-D double-well and 3-D Henon–Heiles models that have been used in previous studies^41,59,60^ (although we note that the exact PES is irrelevant for the results in Figures 6a and 6b). Here, for both CM-DVR and *C*_20_^Tri^(E1; A), the required matrices were calculated for different grid sizes *n*_*g*_, and we report the fraction of unique nonzero elements; in the case of CM-DVR, we chose to count a matrix element with a magnitude of >10^–12^ as being nonzero (note that tests show identical results using a cutoff of 10^–10^).

As expected, the difference in the fractions of nonzero matrix elements between the PS-generated code *C*_20_^Tri^(E1; A) and CM-DVR method is quite clear; for all grid sizes studied, *C*_20_^Tri^(E1; A) requires diagonalization of a workspace matrix, which has typically an order of magnitude fewer nonzero matrix elements than CM-DVR. This is further emphasized in Figure 6c, which shows the ratio of the number of nonzero elements in CM-DVR and *C*_20_^Tri^(E1; A) workspace matrices; as one pushes toward larger grid sizes, the number of nonzero matrix elements in *C*_20_^Tri^(E1; A) is much smaller than in CM-DVR. This is clearly a direct result of the imposed tridiagonal 1-D matrix structure.

The potential advantages of sparsity of *C*_20_^Tri^(E1; A) matrices in larger DVR simulations are further highlighted in Figure 6d. Here, we show the acceleration in computation time, which can be achieved by using *C*_20_^Tri^(E1; A), relative to CM-DVR. Here, for different uniform grid sizes *n*_*g*_ in 2-D and 3-D model PES problems, we determined the time required to calculate the first *n*_*e*_ = 6 eigenvectors of the Hamiltonian matrices from CM-DVR and the workspace matrix from *C*_20_^Tri^(E1; A). Figure 6d shows the ratio of these times, namely
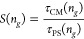
where *S*(*n*_*g*_) is the calculated acceleration for grid size *n*_*g*_, τ_CM_ is the time for CM-DVR eigenvector calculation and τ_PS_ is the time for *C*_20_^Tri^(E1; A) eigenvector calculation. In these calculations, we used sparse-matrix symmetric eigensolvers implemented in the scipy package.^57^

At small grid sizes, there is already a clear acceleration in using *C*_20_^Tri^(E1; A); this relative acceleration increases dramatically as the matrix size increases, as is evident by comparing 2-D and 3-D calculations, and by the trend with grid size. For sparse-matrix manipulations, the computation time would generally be expected to be proportional to the number of nonzero elements in the matrices; it is clear that this rule of thumb broadly holds true in the matrices studied here, with the trend in acceleration (Figure 6d) reflecting the trend in the ratio of nonzero matrix elements (Figure 6c). However, we note that this correspondence is approximate, especially in the 2-D case, likely as a result of additional overheads associated with indexing and sparse-matrix manipulations. For larger problems, with more degrees of freedom and large grids, the order-of-magnitude acceleration, relative to CM-DVR (and other standard DVRs) demonstrated here, might reasonably be expected to increase further still, reflecting the underlying sparsity of *C*_20_^Tri^(E1; A).

**Figure 7:**
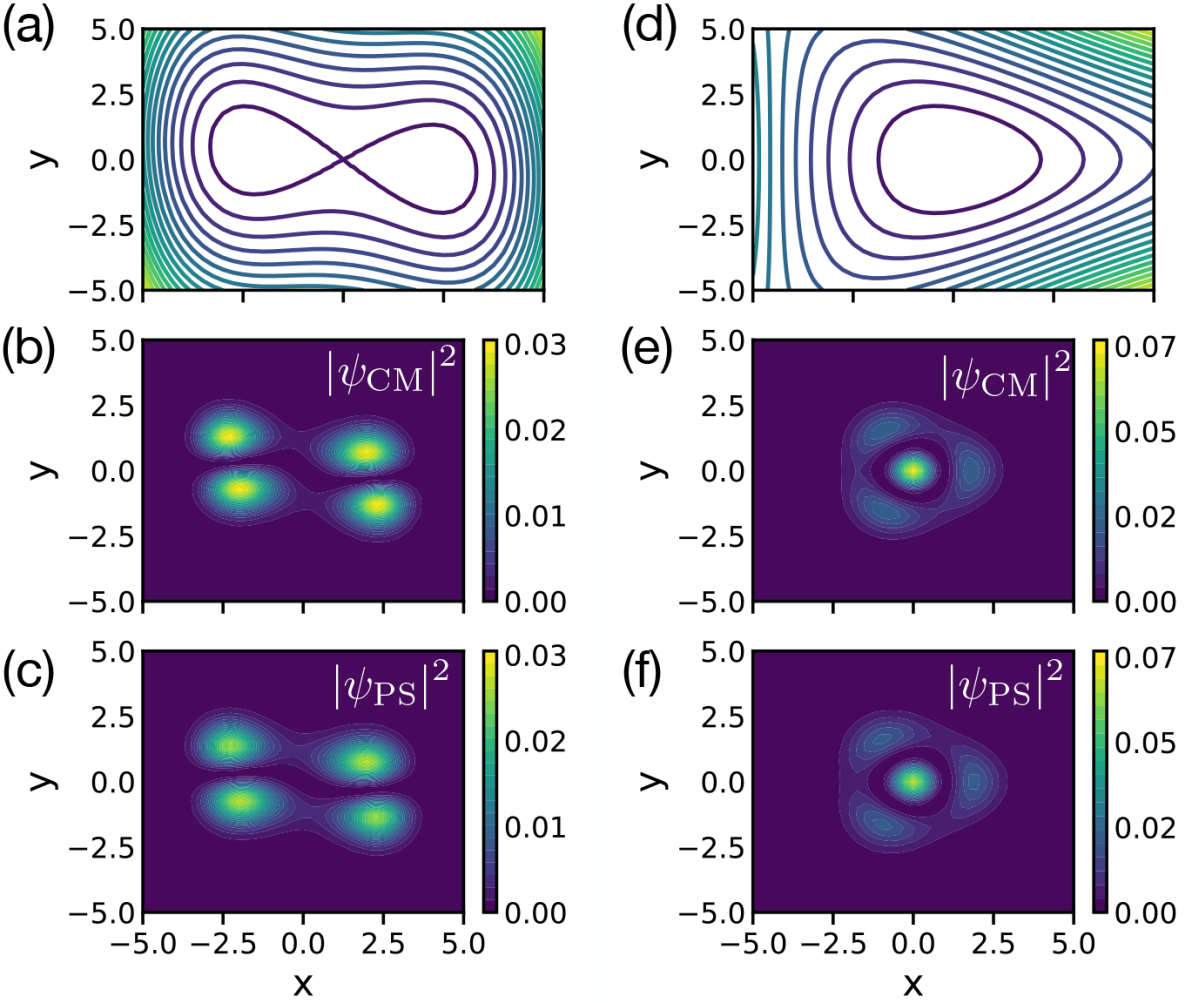
Left column shows (a) the 2-D Henon–Heiles PES (eq 18), (b) the probability density function |ψ_CM_|^2^ of the fourth lowest-energy eigenstate calculated by CM-DVR, and (c) the corresponding probability density function |ψ_PS_|^2^ given by tridiagonal PS-generated matrix *C*_20_^Tri^(E1; A). The right column shows the same plots as panels (a)–(c), but the PES is instead a double-well along *x* coupled to a harmonic oscillator along *y* (see eq 19).

As a final comparison, it is worth verifying that the tridiagonal-based algorithm *C*_20_^Tri^(E1; A) does indeed reproduce eigenvectors for few-dimensional systems (beyond the 1-D systems, which were used in PS optimization). Figure 7 shows the results of 2-D eigenvector calculations using either CM-DVR or *C*_20_^Tri^(E1; A). Here, we consider simulations of two different PESs. Figures 7a–c show the results of simulations of the 2-D Henon–Heiles model given by^41^
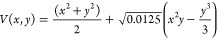
18As a second example, we consider in Figures 7d–f, an additional 2-D PES model describing a double-well potential coupled to a harmonic potential,
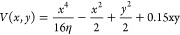
19where η = 1.3544; this double-well PES has been previously been employed as a benchmark system for different quantum dynamics schemes^59,60^ (although the final correlation term is slightly increased here, compared to previous work in order to emphasize this feature in Figure 7).

Figures 7b and 7c show the results of CM-DVR calculations of the *fourth* lowest-energy eigenvector of the two PESs, calculated with a grid size *n*_*g*_ = 41; the results are displayed as |ψ|^2^. Figures 7c and 7f show the same eigenvector, but calculated using *C*_20_^Tri^(E1; A) using the same grid size. The agreement between these two sets of density distributions is clearly very good; this is even more impressive when one considers that the eigenvectors shown are for the fourth lowest-energy allowed state of a 2-D system, whereas the PS optimization only included 1-D information for the first three eigenstates. In particular, for the Henon–Heiles potential, it is clear that *C*_20_^Tri^(E1; A) correctly captures the appearance of the three satellite peaks around the central density peak; in the case of the double-well PES, it is also clear that the appearance of the nodal features of this excited-state wave function are correctly reproduced.

In summary, the results in this section demonstrate that the PS-discovered algorithm *C*_20_^Tri^(E1; A) exhibits a sparser matrix structure compared to CM-DVR; the imposition of the tridiagonal matrix structure leads to significant reduction in the number of nonzero matrix elements in the workspace matrix which is diagonalized to produce the eigenvectors, and this sparsity, in turn, leads to accelerations in computation time, which can be an order of magnitude or more. Note that there is a price to be paid for this improved efficiency in eigenvector prediction using *C*_20_^Tri^(E1; A). In particular, while standard DVR schemes such as CM-DVR yield both the eigenvectors *and* energy eigenvalues from Hamiltonian matrix diagonalization, our PS-generated codes do not directly yield the energy eigenvalues. We have discussed above why this might be the case, namely, the challenge of identifying *Hamiltonian* matrices, compared to the (relative) simplicity of generating alternative matrices, which have the same eigenvectors. However, we have also shown that there is a simple way around this challenge, namely evaluation of energy eigenvalues numerically using the PS-predicted eigenvectors; we have shown that this results in algorithms that effectively demonstrate the same convergence in energy eigenvalues as CM-DVR (with marginally better accuracy). In anticipated applications to larger multidimensional systems and molecular problems, this numerical evaluation of energy will become more challenging, but it is also clear that solutions exist. For example, given that DVR-type algorithms require evaluation of the PES on the grid points as input, it is clear that PES expectation values may be relatively straightforward to evaluate via Monte Carlo methods, using predicted eigenvectors; similarly, local fitting methods, such as the KRR method used here, will also enable evaluation of the KE contribution to the total energy operator. More generally, we note that the same grid-integration problem is encountered (and addressed) in the equations-of-motion employed in MCTDH.^61,62^ Furthermore, we note that recent work in integrating machine learning tools with grid-based wave function methods^19,51−55^ could equally be ported to DVR methods; as a demonstration of the potential reduction in computational expense, we have recently shown that a 12-dimensional (with two electronic states) model of quantum dynamics of pyrazine can be accurately simulated using fewer than 3000 PES (using CASSCF) evaluations as input to a KRR PES.^53,55^ Finally, of course, the search for PS-generated codes that predict both eigenvectors and eigen values will continue.

With regard to future improvements in PS, it is clear that there are a large number of possibilities to be explored beyond the immediate goals of this Article. For example, our recent simulations have demonstrated that nonuniform grids can be used as the basis for our PS scheme. In particular, we have investigated the use of Sobol quasi-random grids^63^ instead of uniform grids; initial PS simulations demonstrate that the accuracy of the resulting algorithms is not quite as good as those based on uniform grids, but improvements in code structure might help. More generally, one could anticipate a DVR-type scheme that works in two stages, first diagonalizing a matrix to give the best choice of grid points for a given PES, and subsequently solving eq 1 using PS-generated codes based on the optimized grids. Furthermore, we note that there is clearly enormous scope for improving both the optimization strategy employed here and the choice of functions. For example, alternative methods such as genetic algorithms could clearly be used as global optimizers to seek out better PS solutions, while methods to evolve better functions or function combinations (such as the ADFs used in genetic programming^43,44^) can also find application here. In short, there is an enormous amount of future possibility, and these initial results serve to highlight the potential of PS in generating new and useful algorithms for quantum chemistry; this has been demonstrated here by focusing on developing a novel DVR-type method with a tridiagonal 1-D workspace matrix, something that has not been previously available, to the best of our knowledge.

## 4 Conclusions

In this Article, we have explored how PS can be used to develop novel grid-based algorithms for calculating wave functions and energies of systems described by the time-independent Schrödinger equation. We have investigated two alternative approaches in PS to generate codes which evaluate the energy, either as matrix eigenvalues or as expectation values. It was found that method E1 (using expectation values) is much more successful, and we have discussed how this is most likely related to the fact that many possible output workspace matrices can have the same set of correct eigenvectors (wave functions), but significantly fewer possess both eigenvectors *and* eigenvalues, which reproduce those of the underlying Hamiltonian matrix.

Perhaps the most important contribution of this Article is the development of a novel DVR-type algorithm, which only employs 1-D matrices with tridiagonal structure; such matrices are extremely sparse, and enable the use of efficient matrix storage and manipulation routines. We have demonstrated the potential of these schemes in calculating eigenvectors for few-dimensional systems; for example, the sparse DVR-like schemes generated by PS here have been demonstrated to result in faster calculation speeds for determining eigenvectors for 2-D and 3-D systems by virtue of employing sparse matrices. This PS approach generates transferrable algorithms that can offer high-quality eigenvector predictions for a broad class of PESs; this is in contrast to previous schemes, such as GP, which focused previously on offering function approximations for single allowed vibrational wave functions on a single defined PES.

Current work is now aimed at improving and developing these methods into general-purpose computer codes that implement these ideas in combination with ab initio PES evaluations for molecular systems; this will, in turn, enable a variety of simulations, including accurate evaluation of molecular vibrational spectra for free-energy calculations and direct wave function dynamics simulations of reactive collisions between few-atom molecules. While the current limit of such full-dimensional quantum simulations using traditional grid-based schemes lies at ∼5 atoms, we hope that further improvements in sparse PS-generated eigenfunction schemes might help push this limit further. We note that there are many remaining important methodological challenges, perhaps most significantly the exploration and refinement of “good” function sets from which to generate new codes; meanwhile, methods such as ADFs, previously employed in the context of GP, are promising, but there is clearly work to do here.

As a final comment, we note that there are also a wide variety of further applications in which PS methods for deriving effective Hamiltonian matrices might be beneficial. In particular, the problem of ab initio electronic structure methods could be a rich domain for future PS applications, seeking new methods which deliver accurate predictions of molecular properties using PS-generated Hamiltonian matrix approximations. This is an ongoing area of research that we hope to expand upon in the near-future.

## Data and Software Availability

Data from Figures 2–6 are available online through the Warwick Research Archive Portal (wrap.warwick.ac.uk/163787).

## Supporting Information Available

Definition of input and internal functions, as well as a discussion of grid-optimized DVR-type algorithms (PDF)
