# Supplementary material for: Program Synthesis of Sparse Algorithms for Wave Function and Energy Prediction in Grid-Based Quantum Simulations
Source: J Chem Theory Comput. 2022 Mar 16;18(4):2462–78. doi: 10.1021/acs.jctc.2c00035 (PMC9009083; doi:10.1021/acs.jctc.2c00035)
Supplement: Supplementary file 1 — ct2c00035_si_001.pdf [file ct2c00035_si_001.pdf]

Supplementary Material: Program synthesis of sparse algorithms for wavefunction and energy prediction in grid-based quantum simulations

Scott Habershon*

Department of Chemistry, University of Warwick, Coventry, CV4 7AL, United Kingdom

E-mail: S.Habershon@warwick.ac.uk

This *Supplementary Material* document contains:

- Tables of input function definitions (Table 1) and internal function definitions (Table 2) used in PS simulations;
- Results and working equations for algorithms optimized for specific grid-sizes.

1 PS function definitions

Table 1: Definition of input vectors $\mathbf{y}$ and matrices $\mathbf{M}$ defined for the $n_i = 11$ input terminals used in PS simulations. Here, $\mathbf{V}$ is the PES evaluted on the input coordinate grid. Explicit elements of the matrix are written as M_{ij}, with M_{ii} representing the matrix diagonal elements.

Input index	Input vector	Input matrix
1	$\mathbf{y} = \mathbf{1}$	$\mathbf{M} = \mathbf{I}$,
2	$\mathbf{y} = \mathbf{1}$	$M_{ij} = 1$, for all i, j
3	$\mathbf{y} = \mathbf{1}$	$M_{ii} = 0$, $M_{ij} = 1$, for $j \neq i$
4	$\mathbf{y} = \mathbf{0}$	$\mathbf{M} = \mathbf{I}$,
5	$\mathbf{y} = \mathbf{0}$	$M_{ij} = 1$, for all i, j
6	$\mathbf{y} = \mathbf{0}$	$M_{ii} = 0$, $M_{ij} = 1$, for $j \neq i$
7	$\mathbf{y} = \mathbf{V}$	$\mathbf{M} = \mathbf{I}$,
8	$\mathbf{y} = \mathbf{V}$	$M_{ij} = 1$, for all i, j
9	$\mathbf{y} = \mathbf{V}$	$M_{ii} = 0$, $M_{ij} = 1$, for $j \neq i$
10	$\mathbf{y} = \mathbf{1}$	$M_{ij} = 1$, if $j = i$ or $j = i \pm 1$, $M_{ij} = 0$ otherwise.
11	$\mathbf{y} = \mathbf{V}$	$M_{ij} = 1$, if $j = i$ or $j = i \pm 1$, $M_{ij} = 0$ otherwise.

Table 2: Internal functions set used in PS simulations. Here, $\mathbf{y}$ indicates the current workspace vector, and $\mathbf{M}$ indicates the workspace matrix. Explicit elements of the vector and matrix are written y_i and M_{ij} respectively, with M_{ii} representing the matrix diagonal elements. The elements x_i represents the position of the i-th grid-point in the uniform grid. Except where indicated, all operations act in an element-wise manner on all entries in the workspace vector or matrix. We note that $I_0(x)$ is the Modified Bessel Function (first kind, zero order), as implemented in `numpy`. Considering all combinations of constants, $c = [m, 2, 3, \pi, 4, L]$, and functions, we have a total of $n_r = 134$ possible operations at each internal code line. We note that this list of functions is clearly not exhaustive and is somewhat arbitrarily chosen based on trial-and-error investigations; however, the results of the main article demonstrate that this set is sufficient to generate new codes which perform very well in eigenfunction prediction when compared to standard DVR schemes.

$\mathbf{y} \to \mathbf{y} \times c$	$\mathbf{y} \to \mathbf{y} + c$	$\mathbf{y} \to \mathbf{y} - c$
$\mathbf{y} \to \mathbf{y}/c$	$\mathbf{y} \to -\mathbf{y}$	$M_{ij} \to \frac{M_{ij}}{(i-j)}, i \neq j$
$M_{ij} \to M_{ij} \times (i-j), i \neq j$	$M_{ij} \to M_{ij} \times (-1)^i, i \neq j$	$M_{ij} \to M_{ij} \times (-1)^j, i \neq j$
$M_{ij} \to \sin(M_{ij})$	$M_{ij} \to \cos(M_{ij})$	$M_{ij} \to M_{ij} \times e^{(x_i - x_j)^2}$
$M_{ij} \to M_{ij} \times e^{-(x_i - x_j)^2}$	$M_{ij} \to e^{M_{ij}}$	$\mathbf{M} \to -\mathbf{M}$
$\mathbf{M} \to \mathbf{M} \times c$	$\mathbf{M} \to \mathbf{M} + c$	$\mathbf{M} \to \mathbf{M} - c$
$\mathbf{M} \to \frac{\mathbf{M}}{c}$	$\mathbf{M} \to \mathbf{M}\mathbf{M}$	$M_{ii} \to M_{ii} \times c$
$M_{ii} \to M_{ii} + c$	$M_{ii} \to M_{ii} - c$	$M_{ii} \to \frac{M_{ii}}{c}$
$\mathbf{M} \to \ln(\mathbf{M})$	$M_{ii} \to \ln(M_{ii})$	$M_{ii} \to M_{ii} \times y_i$
$M_{ii} \to M_{ii} + y_i$	$M_{ii} \to M_{ii} - y_i$	$M_{ii} \to \frac{M_{ii}}{y_i}$
$M_{ij} \to e^{-M_{ij}}$	$M_{ij} \to M_{ij} \times e^{-(x_i - x_j)}$	$M_{ij} \to M_{ij} + e^{-(x_i - x_j)}$
$M_{ij} \to \frac{M_{ij}}{x_i^2}$	$M_{ij} \to \frac{M_{ij}}{x_j^2}$	$M_{ij} \to \frac{M_{ij}}{(-1)^i}, i \neq j$
$M_{ij} \to \frac{M_{ij}}{(-1)^j}, i \neq j$	$M_{ij} \to M_{ij} \times (-1)^i$	$M_{ij} \to \frac{M_{ij}}{(x_i - x_j)}, i \neq j$
$M_{ij} \to M_{ij} \times (x_i - x_j), i \neq j$	$M_{ij} \to \frac{M_{ij}}{(x_i - x_j)^2}, i \neq j$	$M_{ij} \to M_{ij} \times (x_i - x_j)^2, i \neq j$
$M_{ij} \to \frac{M_{ij}}{(x_i - x_j)^3}, i \neq j$	$M_{ij} \to M_{ij} \times (x_i - x_j)^3, i \neq j$	$M_{ij} \to \frac{M_{ij}}{\lvert x_i - x_j \rvert}, i \neq j$
$M_{ij} \to \frac{M_{ij}}{\lvert x_i - x_j \rvert^3}, i \neq j$	$M_{ij} \to \frac{M_{ij}}{(x_i - x_j)^4}, i \neq j$	$M_{ij} \to M_{ij} \times (x_i - x_j)^4, i \neq j$
$M_{ij} \to \sinh(M_{ij})$	$M_{ij} \to \cosh(M_{ij})$	$M_{ij} \to \tanh(M_{ij})$
$M_{ij} \to e^{M_{ij}} - 1$	$M_{ij} \to 2^{M_{ij}}$	$M_{ij} \to \log_{10}(M_{ij})$
$M_{ij} \to \ln(1 + M_{ij})$	$M_{ij} \to I_0(M_{ij})$	$M_{ij} \to \mathrm{sinc}(M_{ij})$
$M_{ij} \to M_{ij}^3$	$M_{ij} \to M_{ij}^4$	$y_i \to \sinh(y_i)$
$y_i \to \cosh(y_i)$	$y_i \to \tanh(y_i)$	$y_i \to e^{y_i} - 1$
$y_i \to 2^{y_i}$	$y_i \to \log_{10}(y_i)$	$y_i \to \ln(1 + y_i)$
$y_i \to I_0(y_i)$	$y_i \to \mathrm{sinc}(y_i)$	$y_i \to y_i^3$
$y_i \to y_i^4$	$M_{ii} \to M_{ii} + V_i$	$M_{ii} \to M_{ii} - V_i$
$M_{ii} \to M_{ii} \times V_i$	$M_{ii} \to \frac{M_{ii}}{V_i}$	

2 Grid-optimized algorithms

It is interesting to ask if the PS-generated algorithms from Table 1 in the main text (which work well across a range of grid-sizes and broadly demonstrate similar convergence to CM-DVR) can be improved upon by seeking separate optimal algorithms for each different grid-size. By removing the constraint that a PS-generated code has to work well for a range of grid-sizes, we might anticipate that generating different algorithms tuned for different grid-sizes might lead to further improvements in accuracy. As such, we performed further PS optimizations but, instead of using a range of grid-sizes in the randomly-generated PES target data, we used single specific grid-sizes. Here, for code size $N = 20$, we performed 100 PS optimizations for fixed grid-sizes $n_g = [13, 15, 19, 21, 31]$ using method E1; beyond fixing the grid-size, the remaining calculation details were the same as noted in sections 2 and 3. At each grid-size, we then selected the best algorithm from each of the 100 PS optimization runs, and further evaluated the performance at the targeted grid-size by calculating E_f for 500 randomly-generated PESs.

Figure 1 shows the results of these simulations as a function of grid-size, compared to the CM-DVR method. (working equations given below). It is found that the grid-targeted algorithms generally improve on the codes generated for grid-ranges (Fig. 4), as might be expected; for example, whereas the average RMS fractional errors obtained by the algorithms in Fig. 4 are around 0.5-1.0% smaller than the corresponding CM-DVR results, in the case grid-optimized algorithms, it is found that the errors are decreased further still, reducing the RMS fractional errors relative to converged CM-DVR by up to ∼9%. Of course, the price paid for this small improvement is the requirement of using different algorithms for different grid-sizes, which is not particularly convenient if one is interested in using PS-generated codes in general analysis of quantum molecular vibrational properties.

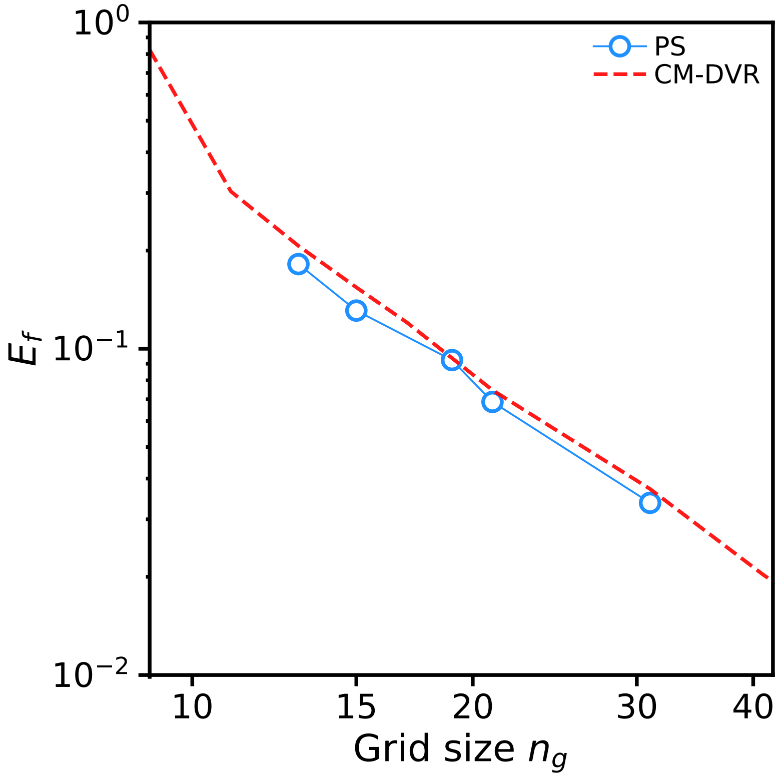

Figure 1: Performance of different grid-targeted algorithms with code size $N = 20$. Here, unique algorithms were generated by PS which were specifically optimized to work for a single grid-size; the PS results here (blue circles) show the RMS fractional errors for five different algorithms targeted to work for different grid-sizes. For comparison, the RMS fractional errors for the Colbert-Miller DVR scheme are also shown (red dashed line). Fractional errors were calculated as in main manuscript for the first $n_e = 3$ eigenstates of 500 randomly-generated PESs; error bars are typically much smaller than the symbol sizes and are not shown for clarity.

2.1 Working equations for grid-optimized algorithms

The following working equations were derived from PS simulations optimized for different grid-sizes:

- $n_g = 13$

$$M_{ij} = (-1)\left[\left(\left(2\tilde{\mathbf{M}}\tilde{\mathbf{M}} - V_i\delta_{ij} + m\right)^3 + 3 + V_i\delta_{ij}\right)e^{-(x_i-x_j)^2} - y_i\delta_{ij}\right] \tag{1}$$

where

$$y_i = 6(V_i - \pi) + 3 \tag{2}$$

and

$$\tilde{M}_{ij} = e^{-(m+1)-4\delta_{ij}} \tag{3}$$

- $n_g = 15$

$$M_{ij} = \begin{cases} \left[(\cos(V_i + \pi) - V_i)\left(\frac{y_i}{2}\right)\right] - \pi, , \text{if } j = i \\ \frac{\cos(1)(-1)^{(i-j)}}{2(i-j)(x_i-x_j)^2}, \text{if } j \neq i \end{cases} \tag{4}$$

where

$$y_i = \sinh\left(\tanh\left[-m\right]\right) \tag{5}$$

- $n_g = 19$

$$M_{ij} = \begin{cases} 4\left(\ln\left[\sin\left(2 - \frac{2}{L}\right)\right] \times 4 + V_i\right), \text{if } j = i \\ \frac{4}{(x_i-x_j)^5}\left(\ln\left[\sin\left(2 - \left(\frac{1}{m(x_i-x_j)^2} + e^{-(x_i-x_j)}\right)\left(\frac{2}{L(x_i-x_j)}\right)\right)\right]\right), \text{if } j \neq i \end{cases} \tag{6}$$

- $n_g = 21$

$$M_{ij} = \begin{cases} 2m(10V_i + 2), \text{if } j = i \\ \frac{2(-1)^{(i+j)}}{4(x_i-x_j)^6}\left(4 + \frac{1}{(i-j)}\right), \text{if } j \neq i \end{cases} \tag{7}$$

- $n_g = 31$

$$M_{ij} = \begin{cases} \frac{\pi V_i}{y_i} \left(\sin\left[\frac{mL+m}{\pi}\right] - 4 \right), \text{if } j = i \\ \frac{\pi \sin(L^4)}{(i-j)}, \text{if } j = i \pm 1 \end{cases} \tag{8}$$

where

$$y_i = 3 + L\left(\tanh(V_i) - L\right) \tag{9}$$
